# Association between cumulative atherogenic index of plasma exposure and risk of myocardial infarction in the general population

**DOI:** 10.1186/s12933-023-01936-y

**Published:** 2023-08-17

**Authors:** Yijun Zhang, Shuohua Chen, Xue Tian, Penglian Wang, Qin Xu, Xue Xia, Xiaoli Zhang, Jing Li, Fen Liu, Shouling Wu, Anxin Wang

**Affiliations:** 1 Department of Neurology, Beijing Tiantan Hospital, Capital Medical University, No. 119 S 4th Ring W Rd, Fengtai District, Beijing, 100070 China; 2 China National Clinical Research Center for Neurological Diseases, Beijing Tiantan Hospital, Capital Medical University, Beijing, China; 3 Department of Clinical Epidemiology and Clinical Trial, Capital Medical University, Beijing, China; 4 Department of Epidemiology and Health Statistics, School of Public Health, Capital Medical University, No.10 Xitoutiao, You’anmen Wai, Fengtai District, Beijing, 100069 China; 5 Beijing Municipal Key Laboratory of Clinical Epidemiology, Beijing, China; 6 Department of Cardiology, Kailuan Hospital, North China University of Science and Technology, Tangshan, China

**Keywords:** Time course, Cumulative exposure, Atherogenic index of plasma, Myocardial infarction

## Abstract

**Background:**

Atherogenic index of plasma (AIP) has been confirmed as a novel marker for myocardial infarction (MI), but few evidence on the long-term AIP and MI risk in general populations. We thus aimed to evaluate the relationships of cumulative exposure to AIP and its accumulation time course with the risk of MI.

**Methods:**

A total of 54,440 participants were enrolled in the Kailuan study. Time-weighted cumulative AIP was calculated as the weighted sum of the mean AIP value for each time interval, then normalized by total exposure duration, the exposure duration was from 2006 to 2010. Duration of high AIP exposure was defined as the duration with high AIP and ranged from 0 to 6 years. The time course of AIP accumulation was categorized by the combination of time-weighted cumulative AIP < or ≥ median (− 0.12) and AIP slope.

**Results:**

After 11.05 years of follow-up, 766 incident MI cases were documented. After adjustment for potential confounders, higher risk of MI was observed in participants with the highest time-weighted cumulative AIP quartile (HR, 1.89; 95% CI 1.47–2.43), the longest exposure duration of high AIP (HR, 1.52; 95% CI 1.18–1.95), and those with high time-weighted cumulative AIP and negative slope (HR, 1.42; 95% CI 1.13–1.79).

**Conclusions:**

Long-term cumulative exposure to AIP and the time course of AIP accumulation increased the risk of MI. High AIP earlier resulted in a greater risk increase than later in life with the same time-weighted cumulative AIP, emphasizing the importance of controlling atherogenic dyslipidemia early in life.

**Supplementary Information:**

The online version contains supplementary material available at 10.1186/s12933-023-01936-y.

## Introduction

Myocardial infarction (MI) is one of the leading causes of mortality from cardiovascular disease (CVD) globally, and the increased prevalence is a burgeoning health threat worldwide [1]. Thus, it is necessary to early identify the population at high risk of MI for public health and clinical practice.

The development of MI is related in large measure to the presence of risk factors, and dyslipidemia is one of the important modifiable risk factors for the development of MI [2, 3]. The common indicators of dyslipidemia including elevated levels of total cholesterol (TC), triglyceride (TG), low-density lipoprotein cholesterol (LDL-C), and the high ratio of LDL-C to high-density lipoprotein cholesterol (HDL-C) both could increase the risk of MI [4–6]. The atherogenic index of plasma (AIP) was first suggested by Dobiásová and Frohlich as a biomarker for plasma atherosclerosis, which is calculated as log (TG/HDL) and reflects both the levels of TG and HDL-C [7]. Recently an increasing number of studies have shown that AIP could be a potential biomarker for the risk of atherosclerosis and CVD [7–10]. AIP not only accurately stands the link between protective and atherogenic lipoproteins but also served as a powerful predictor of atherosclerosis and CVD [11].

Atherosclerosis is one of the major causes of MI which may begin in early life and develop over decades before clinical features appear [12]. AIP is calculated by TG and HDL-C, and these indexes dynamic change in different life stages. However, most recent studies on the association between high AIP levels and CVD risk have focused on AIP levels that were measured at a single time point, and few studies have characterized the long-term exposures to AIP and their implications for CVD risk, and based on special population [13–15]. It is noteworthy that the limited sample size and short follow-up periods, the cumulative affection remains unaccounted for. Furthermore, whether the time course of cumulative AIP accumulation affects the risk of MI is still unclear. Serial elevated levels may be more significant than single elevated measurements. Taken together, it is necessary to evaluate the association between longitudinal AIP and MI risk.

Against this background, this present study investigated to explore the relationship of cumulative exposure to AIP and its accumulation time course with the risk of MI.

## Methods

### Study population

The study enrolled participants from the Kailuan study, which was an ongoing, prospective, cohort study conducted in the Kailuan community in Tangshan City, China. Details of the study design and methods for the Kailuan study have been reported previously [16, 17]. From June 2006 to October 2007, 101,510 individuals (81,110 men and 20,400 women) aged 18 years or above were enrolled and biennially attended follow-up visits including the questionnaire survey, physical examination, and routine laboratory tests. The Kailuan study was performed according to the guidelines of the Helsinki Declaration and was approved by the Ethics Committee of Kailuan Hospital (approval number: 2006e05) and Beijing Tiantan Hospital (approval number: 2010–014-01). Each participant provided their written informed consent before participating in the study.

In this current study, we first excluded 15,123 participants with less than 3 health examinations from 2006 to 2010, then we excluded 30,539 participants with missing data of TG and HDL-C. We further excluded 1,408 participants who developed MI or death from 2006 to 2010. Finally, a total of 54,440 participants were included in the present study. Additional file 1: Figure S1 displays the flowchart of participant enrollment.

### Data collection and definitions

The demographic characteristics and lifestyle behaviors factors, such as age, sex, educational levels, income, smoking status, drinking status, physical exercise habits as well as past medical and medication histories, were obtained through face-to-face standardized questionnaires. Educational levels were classified as above middle school or not. Income level was categorized as > 800 and ≤ 800 yuan per month. Smoking and drinking status were classified as yes or no. Active physical exercise was defined as ≥ 80 min per week. Body mass index (BMI) was calculated as weight (kg)/ height (m) [2]. Systolic blood pressure (SBP) and diastolic blood pressure (DBP) were calculated as the average of three readings when the participants were in the seated position using a mercury sphygmomanometer. Blood was obtained from the antecubital vein early in the morning after 12 h fast, and all the plasma samples were assessed using an auto-analyzer (Hitachi 747, Tokyo, Japan) at the central laboratory of Kailuan Hospital. Fasting plasma glucose (FPG) was measured using the hexokinase/glucose-6-phosphate dehydrogenase method with the coefficient of variation using blind quality control specimens < 2.0%. We used the creatinine-based Chronic Kidney Disease Epidemiological Collaboration (CKD-EPI 2009) equation to calculate the estimated glomerular filtration rate (eGFR) [18]. Serum TC, TG, LDL-C, and high-density lipoprotein cholesterol (HDL-C) were measured with the enzymatic colorimetric method. Plasma high-sensitivity C-reactive protein (hs-CRP) concentrations were measured with a high-sensitivity particle-enhanced immunonephelometry assay (Cias Latex CRP-H, Kanto Chemical Co. Inc.). Hyperlipidemia was defined as a history of hyperlipidemia, or taking any lipid-lowering drugs, or TC ≥ 5.17 mmol/L [19].

### Calculation of time-weighted cumulative AIP, duration of high AIP exposure and time course of AIP accumulation

The AIP was mathematically derived from log (TG/HDL-C), as previously reported [7]. Time-weighted cumulative AIP was calculated as the weighted sum of the mean AIP value for each time interval, then normalized by total exposure duration, the exposure duration was from 2006 to 2010 and referenced to the time-weighted cumulative blood pressure [20]. The formula of time-weighted cumulative AIP was [(AIP_2006_ + AIP_2008_)/2 × time_2006-2008_ + (AIP_2008_ + AIP_2010_)/2 × time_2008-2010_]/time_2006-2010_, where AIP_2006_, AIP_2008_, and AIP_2010_ indicated the first (2006–2007), second (2008–2009) and third (2009–2010) examinations, time_2006-2008_ and time_2008-2010_ indicated the time intervals between two consecutive visits in years, and time_2006-2010_ indicated the time intervals between first to third visits in years. The means of time_2006-2008_ and time_2008-2010_ were 2.08 and 1.98 years, and the mean of time_2006-2010_ was 3.96 years. The participants were stratified by quartiles of time-weighted cumulative AIP: Q1 group, ≤ − 0.50 (as reference group), Q2 group, − 0.50 to − 0.12, Q3 group, − 0.12 to 0.28, and Q4 group, ≥ 0.28.

High AIP was defined as AIP in the highest quartile [21]. The duration of high AIP exposure was defined as the duration with high AIP among the first three examinations, quantified as 0 year (never, as reference group), 2 years (once), 4 years (twice), and 6 years (all three study visits).

The time course of AIP accumulation was defined by time-weighted cumulative AIP joint AIP slope, referenced to the time course of serum uric acid accumulation [22]. The time-weighted cumulative AIP was categorized as low and high by median, the median of time-weighted cumulative AIP was − 0.12. Using the method of linear regression to estimate AIP level versus time from 2006 to 2010, increase in AIP over time with a positive slope, and decrease in AIP over time with a negative slope. Then the participants were stratified into four groups: low time-weighted cumulative AIP with positive slope (as reference group), low time-weighted cumulative AIP with negative slope, high time-weighted cumulative AIP with positive slope, high time-weighted cumulative AIP with negative slope, respectively.

### Ascertainment of MI

The primary outcome was the first occurrence of MI (International Classification of Disease-10 [ICD-10]: I21) during follow-up, either fatal or nonfatal. The database of MI diagnosis was confirmed from the Municipal Social Insurance Institution and Hospital Discharge Register and was updated annually during the follow-up period. The diagnosis of MI was determined by the patient’s clinical symptoms, electrocardiogram, and dynamic changes of myocardial enzyme following the World Health Organization’s Multinational Monitoring of Trends and Determinants in Cardiovascular Disease criteria [23]. Follow-up ended at the first onset of MI, all-cause death, or at the end of follow-up on 31 December 2021, whichever came first.

### Statistical analysis

Baseline characteristics were described by mean (standard deviation, SD) or median (interquartile range, IQR) or number (proportions), as appropriate. The group differences were compared using one-way analysis or the Wilcoxon rank-sum test for continuous variables, and the chi-square test for categorical variables. Person-years at risk were computed from the date of baseline until the date of end follow-up or the date of onset of MI, death, whichever came first. The cumulative incidences of new-onset MI for each group were calculated using the Kaplan–Meier methods and compared by log-rank test.

Unadjusted and adjusted Cox proportional hazards regression models were used to estimate the relationships of time-weighted cumulative AIP, exposure duration of high AIP, and the time course of AIP accumulation with MI incidence risk by calculating the hazard ratios (HRs) and 95% confidence intervals (95% CIs). There were four Cox proportional hazards regression models: Model 1 was unadjusted; Model 2 was adjusted for age and sex at baseline; Model 3 was further adjusted for education, smoking status, drinking status, BMI, SBP, DBP, FBG, history of hypertension, hyperlipidemia, diabetes at baseline; Model 4 was further adjusted for hs-CRP, eGRF, TC, LDL-C, antihypertensive drugs, antidiabetic drugs, and lipid-lowering drugs at baseline. The *P* values for trend were computed using quartiles of time-weighted cumulative AIP, duration of high AIP exposure, and the time course of AIP accumulation as ordinal variables. Restricted cubic spline (RCS) with 5 knots (at the 5th, 25th, 50th, 75th, and 95th percentiles) was used to analyze the effect of time-weighted cumulative AIP on MI as a continuous variable.

To evaluate the robustness of the association of time-weighted cumulative AIP, duration of high AIP exposure, and the time course of AIP accumulation with the risk of MI, sensitivity analyses were applied, adjusted for covariables in Model 4 and further considering non-MI related death as competing event during the follow-up visits. Stratified analyses according to baseline age (≤ 65 vs. > 65 years), sex (female vs. male), BMI (< 24 vs. ≥ 24 kg/m [2]), hypertension (no vs. yes), antihypertension drugs (no vs. yes), diabetes (no vs. yes), antidiabetic drugs (no vs. yes), hyperlipidemia (no vs. yes), lipid-lowering drugs (no vs. yes), and baseline LDL-C level (≤ 2.6 mmol/L vs. > 2.6 mmol/L) were used to examine the consistency of the effect of time-weighted cumulative AIP, duration of high AIP exposure and the time course of AIP accumulation with the risk of MI. Additionally, we compared the incremental predictive value of time-weighted cumulative AIP and other lipid indexes beyond conventional risk factors by C statistics, integrated discrimination improvement (IDI), and net reclassification index (NRI).

All statistical analyses were done with SAS 9.4 (SAS Institute, Cary, NC, USA). All *P* values were two-sided, and *P* < 0.05 was considered statistically significant.

## Results

### Baseline characteristics

A total of 54,440 participants were included, of which the media age was 49.58 years and 76.51% were males. The average time-weighted cumulative AIP was -0.09 ± 0.61. The baseline characteristics of participants according to quartiles of time-weighted cumulative AIP were shown in Table 1. Compared with the lowest time-weighted cumulative AIP level, participants with higher time-weighted cumulative AIP levels were more likely to be males, less well educated, lower income, physically inactive, current smokers, and current drinkers, a higher prevalence of hypertension, hyperlipidemias, diabetes, more likely to take antihypertensive drugs, antidiabetic drugs, and lipid-lowering drugs, had higher BMI, blood pressures, FPG, hs-CRP, eGFR, TC, and LDL-C levels. When participants were categorized by the duration of high AIP exposure, and the time course of AIP accumulation, similar trends of baseline characteristics were observed in participants with 6 years of exposure and high time-weighted cumulative AIP with negative slope (Additional file 1: Tables S1, S2). There were 47,070 individuals excluded from the Kailuan cohort in this study. A comparison of baseline characteristics between included and excluded individuals was given in Additional file 1: Table S3.

**Table 1:** Baseline characteristics of participants according to quartiles of time-weighted cumulative AIP

Characteristics	Overall	Q1 (≤ − 0.50)	Q2 (− 0.50 to -0.12)	Q3 (− 0.12 to 0.28)	Q4 (≥ 0.28)	*P* value
No. of participants	54,440	13,610	13,610	13,610	13,610	
Age, year	49.58 (41.87–56.57)	49.82 (41.70–56.98)	49.85 (42.06–56.95)	49.71 (41.91–56.72)	48.93 (41.81–55.52)	< 0.0001
Male, n (%)	41,652 (76.51)	9388 (68.98)	10,135 (74.47)	10,798 (79.34)	11,331 (83.25)	< 0.0001
High school or above, n (%)	4327 (7.95)	1311 (9.63)	1013 (7.44)	937 (6.88)	1066 (7.83)	< 0.0001
Income > 800 RMB/month, n (%)	8198 (15.06)	2136 (15.69)	2008 (14.75)	1926 (14.15)	2128 (15.64)	0.001
Active physical activity, n (%)	47,790 (87.78)	12,091 (88.84)	11,931 (87.66)	11,908 (87.49)	11,860 (87.14)	0.0001
Current smoking, n (%)	18,391 (33.78)	4190 (30.79)	4392 (32.27)	4514 (33.17)	5295 (38.91)	< 0.0001
Current drinking, n (%)	21,150 (38.85)	5092 (37.41)	4998 (36.72)	5129 (37.69)	5931 (43.58)	< 0.0001
BMI, kg/m^2^	24.91 (22.66–27.25)	23.12 (21.11–25.21)	24.51 (22.41–26.76)	25.40 (23.42–27.61)	26.40 (24.46–28.44)	< 0.0001
SBP, mmHg	125.30 (115–140)	120 (110–134)	124 (113.30–140)	129.30 (119.30–140)	130 (120–141.30)	< 0.0001
DBP, mmHg	80 (76.70–90)	80 (70.70–84.70)	80 (75–90)	80 (79.30–90)	81.30 (80–90)	< 0.0001
Hypertension, n (%)	21,475 (39.45)	3888 (28.57)	5130 (37.69)	6059 (44.52)	6398 (47.01)	< 0.0001
Hyperlipidemia, n (%)	4456 (8.19)	633 (4.65)	935 (6.87)	1265 (9.29)	1623 (11.93)	< 0.0001
Diabetes Mellitus, n (%)	18,705 (34.36)	1869 (13.73)	2864 (21.04)	4681 (34.39)	9291 (68.27)	< 0.0001
Antihypertensive drugs, n (%)	5201 (9.55)	829 (6.09)	1153 (8.47)	1457 (10.71)	1762 (12.95)	< 0.0001
Antidiabetic drugs, n (%)	1128 (2.07)	200 (1.47)	254 (1.87)	302 (2.22)	372 (2.73)	< 0.0001
Lipid-lowering drugs, n (%)	486 (0.89)	68 (0.50)	116 (0.85)	118 (0.87)	184 (1.35)	< 0.0001
FPG, mmol/L	5.10 (4.64–5.66)	4.92 (4.50–5.40)	5.08 (4.63–5.60)	5.12 (4.69–5.73)	5.24 (4.76–5.90)	< 0.0001
hs-CRP, mg/L	0.78 (0.30–2.13)	0.58 (0.20–1.72)	0.72 (0.29–2.00)	0.80 (0.31–2.20)	1.00 (0.41–2.56)	< 0.0001
eGFR, mL/min/1.73m^2^	82.7 (69.42–97.18)	84.06 (71.56–97.51)	82.75 (69.17–97.39)	80.93 (67.44–96.38)	82.92 (69.55–97.39)	< 0.0001
TC, mmol/L	4.90 (4.26–5.56)	4.75 (4.19–5.38)	4.89 (4.28–5.52)	4.92 (4.25–5.60)	5.04 (4.35–5.77)	< 0.0001
LDL-C, mmol/L	2.30 (1.77–2.79)	2.11 (1.59–2.68)	2.32 (1.85–2.80)	2.40 (1.93–2.82)	2.30 (1.76–2.80)	< 0.0001

Continuous variables were reported as median (IQR). Categorical variables were reported as N(%)

*AIP* atherogenic index of plasma, *BMI* body mass index, *SBP* systolic blood pressure, *DBP* diastolic blood pressure, *FPG* fasting plasma glucose, hs-*CRP* high-sensitivity C-reactive protein, *eGFR* estimated glomerular filtration rate, *TC* total cholesterol, *LDL-C* low-density lipoprotein cholesterol

### Association between time-weighted cumulative AIP, duration of high AIP exposure, and time course of AIP accumulation with the risk of MI

**Fig. 1:**
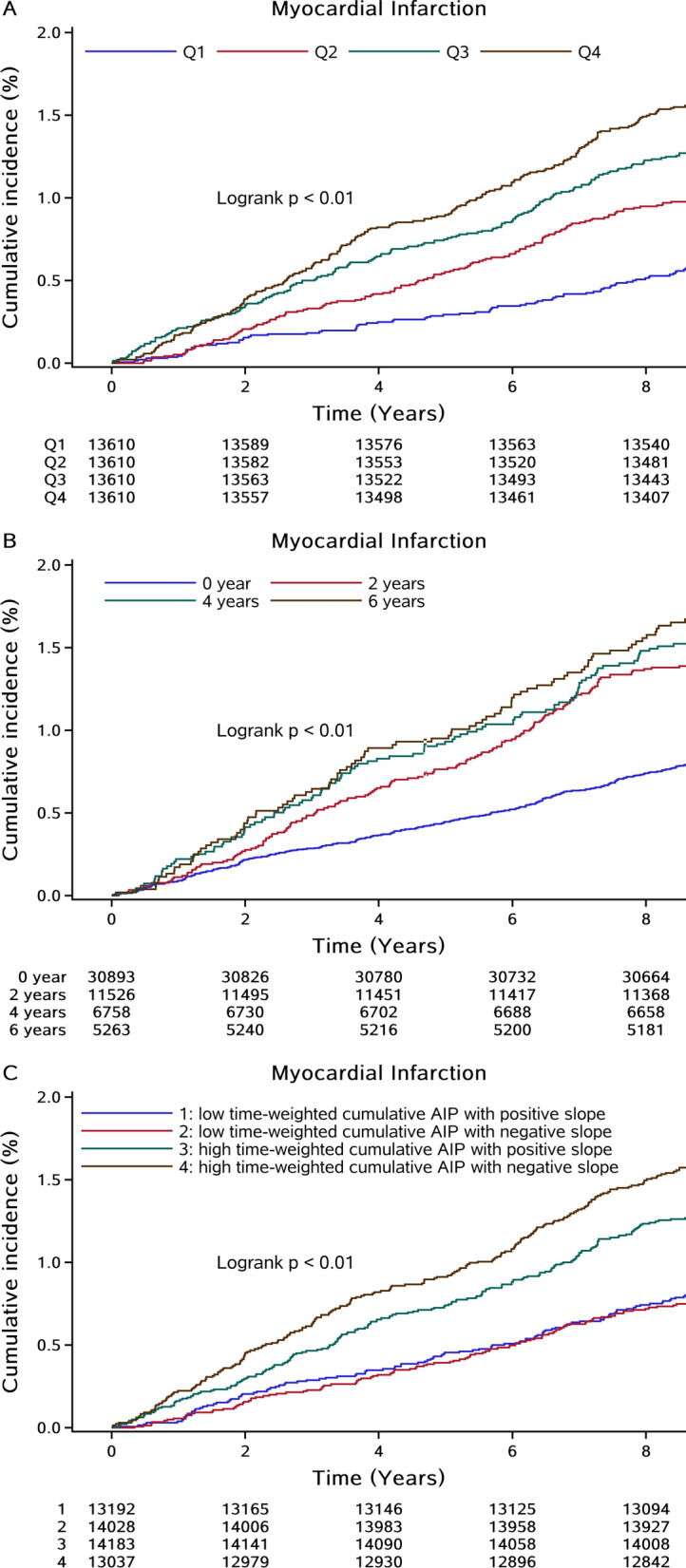
Kaplan–Meier curves of MI incidence rate by quartiles of time-weighted cumulative AIP duration of high AIP exposure, and time course of AIP accumulation. *AIP* atherogenic index of plasma, *MI* myocardial infarction

**Fig. 2:**
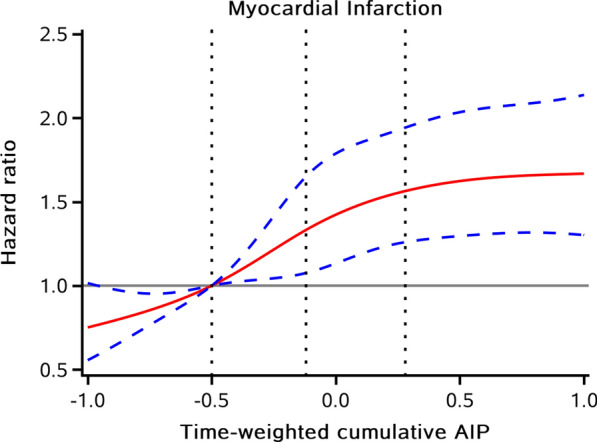
Multivariable-adjusted hazard ratios for MI based on restricted cubic spines with 5 knots at 5th, 25th, 50th, 75th, and 95th percentiles of time-weighted cumulative AIP, and the red line represents references for hazard ratios, and blue lines represent 95% confidence interval. *AIP* atherogenic index of plasma, *MI* myocardial infarction. Adjusted for age, sex, education, income, smoking status, drinking status, body mass index, systolic blood pressure, fasting plasma glucose, high sensitivity C-reactive protein, estimated glomerular filtration rate, total cholesterol, low-density lipoprotein cholesterol level, history of hypertension, hyperlipidemia, diabetes, antihypertensive drugs, antidiabetic drugs, and lipid-lowering drugs at baseline

After a median follow-up of 11.05 years, 766 incident MI cases were documented among 54,440 participants. The incidence rate of MI in each quartile of time-weighted cumulative AIP groups was 0.72 (95% CI 0.60–0.88), 1.13 (95% CI 0.97–1.31), 1.52 (95% CI 1.33–1.73) and 1.79 (95% CI 1.59–2.02) per 1000 person-years, respectively. As shown in Fig. 1A, the Kaplan–Meier curve indicated a stepwise increase in the incidence of MI across the patterns of time-weighted cumulative AIP (*P* < 0.01 for the log-rank test). The associations of time-weighted cumulative AIP exposure measures with MI risk were presented in Table 2. Compared with the lowest quartile group, the risk of MI was significantly increased in higher quartile groups, with the HRs and 95%CIs were 1.39 (1.09–1.78), 1.73 (1.36–2.19), and 1.89 (1.47–2.43) respectively in Q2, Q3, and Q4 group after adjustment for traditional risk factors (*P* for trend < 0.001). Multivariable-adjusted RCS showed *J*-shaped affection of time-weighted cumulative AIP on MI risk when considered as a continuous variable (Fig. 2).

**Table 2:** HR and 95% CI for the risk of myocardial infarction stratified by time-weighted cumulative AIP, duration of high AIP exposure and time course of AIP accumulation

Index	Case, n (%)	Incidence rate, per 1000 person-years	Model 1 HR (95% CI)	Model 2 HR (95% CI)	Model 3 HR (95% CI)	Model 4 HR (95% CI)	Sensitivity analysis^a^ HR (95% CI)
Time-weighted cumulative AIP							
Q1 (≤ -0.50)	108 (0.79)	0.72 (0.60–0.88)	Reference	Reference	Reference	Reference	Reference
Q2 (-0.50 to -0.12)	168 (1.23)	1.13 (0.97–1.31)	1.56 (1.22–1.99)	1.56 (1.23–1.99)	1.40 (1.09–1.78)	1.39 (1.09–1.78)	1.38 (1.08–1.76)
Q3 (-0.12 to 0.28)	225 (1.65)	1.52 (1.33–1.73)	2.09 (1.66–2.63)	2.11 (1.68–2.66)	1.71 (1.35–2.17)	1.73 (1.36–2.19)	1.72 (1.35–2.18)
Q4 (≥ 0.28)	265 (1.95)	1.79 (1.59–2.02)	2.47 (1.97–3.09)	2.62 (2.09–3.28)	1.86 (1.45–2.39)	1.89 (1.47–2.43)	1.91 (1.49–2.45)
*P* for trend			< 0.0001	< 0.0001	< 0.0001	< 0.0001	< 0.0001
Duration of high AIP exposure^b^							
0 year	328 (1.06)	0.97 (0.87–1.08)	Reference	Reference	Reference	Reference	Reference
2 years	199 (1.73)	1.58 (1.38–1.82)	1.63 (1.36–1.94)	1.69 (1.42–2.02)	1.45 (1.20–1.74)	1.49 (1.24–1.79)	1.46 (1.21–1.76)
4 years	133 (1.97)	1.81 (1.53–2.14)	1.86 (1.52–2.27)	1.95 (1.59–2.39)	1.52 (1.22–1.90)	1.57 (1.26–1.96)	1.58 (1.27–1.97)
6 years	106 (2.01)	1.85 (1.53–2.24)	1.91 (1.53–2.37)	2.04 (1.63–2.54)	1.48 (1.15–1.90)	1.52 (1.18–1.95)	1.54 (1.20–2.00)
*P* for trend			< 0.0001	< 0.0001	< 0.0001	< 0.0001	< 0.0001
Time course of AIP accumulation^c^							
Low time-weighted cumulative AIP with positive slope	135 (1.02)	0.93 (0.79–1.11)	Reference	Reference	Reference	Reference	Reference
Low time-weighted cumulative AIP with negative slope	141 (1.01)	0.92 (0.78–1.09)	0.99 (0.78–1.25)	0.90 (0.71–1.15)	0.88 (0.69–1.11)	0.89 (0.70–1.13)	0.90 (0.71–1.14)
High time-weighted cumulative AIP with positive slope	233 (1.64)	1.51 (1.33–1.71)	1.61 (1.31–1.99)	1.69 (1.36–2.09)	1.37 (1.10–1.71)	1.36 (1.09–1.69)	1.37 (1.10–1.71)
High time-weighted cumulative AIP with negative slope	257 (1.97)	1.81 (1.60–2.05)	1.94 (1.58–2.39)	1.80 (1.46–2.22)	1.34 (1.07–1.68)	1.42 (1.13–1.79)	1.44 (1.14–1.81)
*P* for trend			< 0.0001	< 0.0001	< 0.0001	< 0.0001	0.0003

*AIP* atherogenic index of plasma

Model 1: unadjusted

Model 2: adjusted for age and sex

Model 3: Model 2 plus further adjusted for education, income, smoking status, drinking status, body mass index, systolic blood pressure, diastolic blood pressure, fasting plasma glucose, history of hypertension, hyperlipidemia, diabetes at baseline

Model 4: Model 3 plus further adjusted for high sensitivity C-reactive protein, estimated glomerular filtration rate, total cholesterol, low-density lipoprotein cholesterol level, antihypertensive drugs, antidiabetic drugs, and lipid-lowering drugs at baseline

^a^Sensitivity analysis was adjusted for covariables in Model 4 and further considering death as competing event

^b^High AIP was defined as AIP in the highest quartile

^c^Median of cumulative AIP was − 0.12

Figure 1B showed a progressively increasing risk of the incidence rate of MI and MI risks with a greater duration of high AIP exposure (*P* < 0.01 for the log-rank test). After adjustment for potential confounders, compared with the shortest cumulative duration of high AIP exposure (0 year), the risk of MI was significantly higher in those in 2 years group, 4 years group, and 6 years group, with the HRs and 95%CIs were 1.49 (1.24–1.79), 1.57 (1.26–1.96), and 1.52 (1.18–1.95), respectively (*P* for trend < 0.001; Table 2).

Highest incidence rate of MI was observed in participants with high time-weighted cumulative AIP and negative slope of AIP (*P* < 0.01 for the log-rank test; Fig. 1C). When considering the combined effect of time-weighted cumulative and slope of AIP, the results showed that individuals with high time-weighted cumulative AIP and negative slope had the highest risk of MI (HR, 1.42; 95% CI 1.13–1.79; Table 2) among the 4 groups.

### Additional analyses

**Fig. 3:**
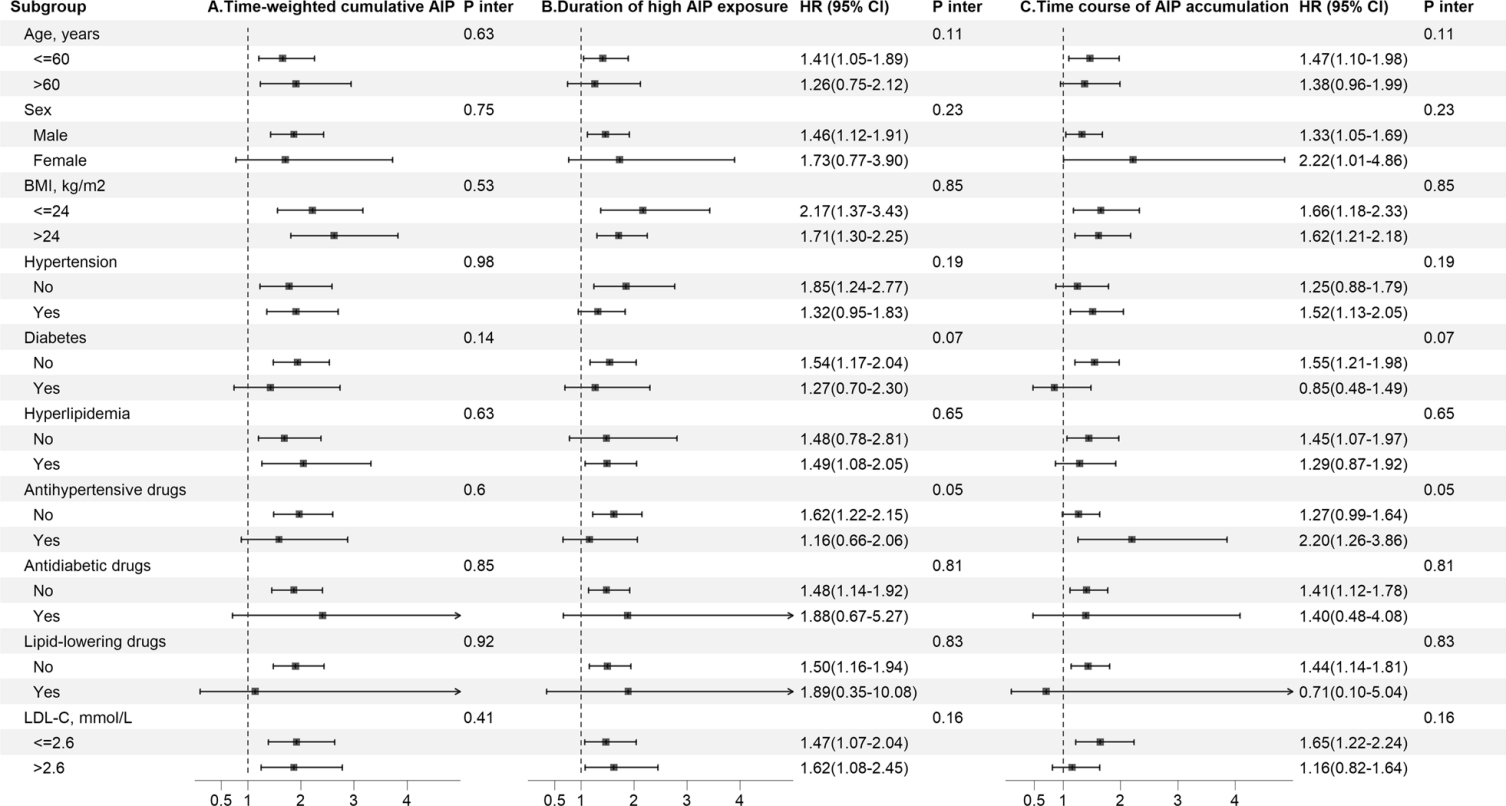
Subgroup analyses for the association with A time-weighted AIP, B duration of high AIP exposure, and time course of AIP accumulation with risk of myocardial infarction. *AIP* atherogenic index of plasma, *CI* confidence interval, *HR* hazard ratio, *BMI* body mass index. Adjusted for age, sex, education, income, smoking status, drinking status, body mass index, systolic blood pressure, diastolic blood pressure, fasting plasma glucose, history of hypertension, hyperlipidemia, diabetes, high sensitivity C-reactive protein, estimated glomerular filtration rate, total cholesterol, low-density lipoprotein cholesterol level, antihypertensive drugs, antidiabetic drugs, and lipid-lowering drugs at baseline other than the variable for stratification

Considering non-MI related death as a competing event and adjusting for covariables in Model 4, the results of sensitivity analyses were consistent with the main analyses (Table 2). In the subgroup analyses, the association between higher quartiles of time-weighted cumulative AIP with risk of MI was consistent and significant across subgroups generally (*P* for interaction > 0.05 for all; Fig. 3 and Additional file 1: Table S4). Similar results were obtained for the subgroup analysis between the duration of high AIP exposure or time course of AIP accumulation and risk of MI (Fig. 3, Additional file 1: Tables S5, S6).

### Incremental predictive value of time-weighted cumulative AIP

We evaluated whether time-weighted cumulative AIP had an increment effect on the predictive value for MI (Additional file 1: Table S7). After the conventional risk model added time-weighted cumulative AIP, the C-statistics improved from 0.73 to 0.74 (P = 0.002), while the IDI of 0.05% (95% CI 0.01–0.08; P = 0.009), and the NRI of 17.64% (95% CI 10.52–24.75; P < 0.0001).

## Discussion

In this prospective cohort study of 54,440 individuals from the Kailuan study, we found that higher cumulative and longer exposure duration of AIP increased the risk of MI. It's worth noting that even though time-weighted cumulative AIP was the same, acquired high AIP earlier caused a greater risk of MI increased than later in life. In addition, this risk was not attenuated when considering all-cause mortality as a competing event. These findings emphasized the importance of monitoring long-term AIP and lowering AIP started early in life. In addition to cumulative AIP being a risk factor for MI, our study highlighted the importance of optimal AIP levels early in life. What’s more, the results only suggested elevated AIP in early life caused a persistent increase in later MI risk, but not referred that both lowering TG and raising HDL-C had no primary prevention benefits.

The significance of TG in CVD has been emphasized in recent clinical practice [24–26]. Additionally, recent data have shown that an increase in HDL-C levels is associated with a lower risk of CVD beyond LDL-C levels [27–30]. The ratio of TG to HDL-C has been suggested to be more useful to reflect plasma atherogenicity than individual lipid values, considering the complex interactions of lipoprotein metabolism [21]. Previous studies have found that a higher level of AIP increased the risk of developing CVD. Elevated AIP was confirmed as an independent and positive risk factor of CVD among non-diabetic hypertensive older adults [31]. There was evidence that AIP was superior to the traditional lipid indices to independently predict CAD risk among Chinese postmenopausal women [32]. A nationwide population-based cohort study showed baseline AIP was significantly associated with CVD risk, with the HR of the highest quartile being 1.278 (95CI %, 1.209–1.350) [33]. In our study, compared with the lowest quartile of time-weighted cumulative AIP, the MI risk in the highest quartile increased by 89% (HR, 1.89; 95CI %, 1.47–2.43), which is consistent with the previous study. However, there are few evidence based on the general population, and most past studies assessed AIP at a single time point [13, 14, 33, 34] and ignored the longitudinal variations of AIP over time, which would result in potential regression dilution bias and may affect the accuracy of the results.

To the best of our knowledge, few cohort studies have explored AIP by repeated measurements analysis. Another Kailuan study showed that cumulative AIP modified the risk of type 2 diabetes (T2DM) [35], but this study didn’t consider the follow-up period differences in individuals. In the secondary analysis of the China Health and Retirement Longitudinal Study, the differences in AIP between baseline and a subsequent examination were used to predict the risk of T2DM, and the results showed that maintained-high AIP, high-to-low AIP, and low-to-high AIP were associated with the development of T2DM in middle‑aged and older Chinese [36].

Hyperlipidemia may begin in early life cause the occurrence and development of atherosclerosis, then increase the risk of MI in the long term [37–39]. Therefore, it’s necessary to evaluate cumulative exposure intensity and duration of high cholesterol. In this study, we explored the associations between the long-term AIP level and MI. Our study suggested that the risk of MI increased with higher cumulative, longer exposure duration, and earlier exposure to high AIP. These results highlight the need for monitoring cholesterol levels in the long term and starting lower cholesterol in early life. Previous research was more based on type 2 diabetes [40–42] or non-diabetes population [43]. Considering the affection of diabetes or other chronic diseases, then we further performed subgroup analyses and found that high cumulative AIP exposure was significantly associated with the risk of MI in various subgroups generally. The previous studies showed that AIP has a significant association with cardiovascular diseases in diabetes [15, 33, 36]. Kim SH et al. reported that the CVD risk was higher in diabetics than non-diabetics, but this study didn’t provide the interaction effect in AIP and diabetics, thus the risk difference between the two groups was unknown [33]. Fu L et al. focused on the single measurement AIP among patients with type 2 diabetes mellitus [15], and Yi Q et al. explore the longitudinal effect of AIP on type 2 diabetes in middle-aged and older Chinese [36]. Other studies showed AIP was also associated with cardiovascular diseases in non-diabetic [14, 31]. In our study, we focused on the longitudinal effect of AIP among the general population, the associations had the same trends in diabetes or non-diabetes but no statistical significance in diabetes. More importantly, there was no interaction between AIP and diabetes mellitus, which means no difference between the two subgroups.

Our study has important implications for MI prevention. The cumulative AIP exposure might help identify individuals at high risk for developing MI in a large and long-term follow-up cohort. For the general population, maintaining an appropriate level of TG and HDL-C within the desirable range and better control of cumulative AIP is important for controlling chronic diseases. Strengths of the current study included the use of its prospective cohort design, large sample size, a long follow-up for MI events, and repeated measurements of multiple laboratory variables. However, our investigation has several limitations.

Firstly, this was an observational study, and causality cannot be demonstrated, though we adjusted several confounding factors such as demographic characteristics, lifestyle, disease history and so on. There might be others included in the study that we could not control for, so our findings need to be confirmed in future studies. Secondly, the current findings are based solely on data from Northern Chinese coal miners, who were mainly males, so the findings may not be generalizable to other populations. However, on the other hand, the population in our study was quite homogeneous, which to some extent made our findings more reliable. Furthermore, the data on demographic characteristics, lifestyle behaviors factors, and medical and medical histories in this study, were obtained through self-reported questionnaires, which are affected by systematic errors and thus must be interpreted with caution. In this regard, we adjusted many potential confounders, we also added sensitivity analysis and subgroups analysis, and the results remain stable. Further prospective studies are needed to replicate our results.

## Conclusions

In conclusion, the study showed that long-term cumulative exposure of AIP, and the time course of AIP accumulation increased the risk of MI. The same time-weighted cumulative AIP level acquired high AIP earlier in life resulting in a greater risk increase than later in life. Assessment and management of atherogenic dyslipidemia early in life are instrumental for MI prevention.

### Supplementary Information

**Additional file 1: Figure** **S1.** The flowchart of the study. **Table S1.** Baseline characteristics of participants stratified by duration of high AIP exposure. **Table S2.** Baseline characteristics of participants stratified by time course of AIP accumulation. **Table S3.** Baseline characteristics of excluded and included participants. **Table S4.** Subgroup analysis for the association between time-weighted cumulative AIP and risk of myocardial infarction. **Table S5.** Subgroup analysis for the association between duration of high AIP exposure and risk of myocardial infarction. **Table S6.** Subgroup analysis for the association between time course of AIP accumulation and risk of myocardial infarction. **Table S7.** Reclassification and discrimination statistics for risk of MI by time-weighted cumulative AIP.

## Data Availability

The datasets used and/or analyzed during the current study are available from the corresponding author upon reasonable request.
