# Supplementary material for: Association between cumulative atherogenic index of plasma exposure and risk of myocardial infarction in the general population
Source: Cardiovasc Diabetol. 2023 Aug 17;22:210. doi: 10.1186/s12933-023-01936-y (PMC10436658; doi:10.1186/s12933-023-01936-y)
Supplement: Supplementary file 1 — Additional file 1: Figure S1. The flowchart of the study. Table S1. Baseline characteristics of participants stratified by duration of high AIP exposure. Table S2. Baseline characteristics of participants stratified by time course of AIP accumulation. Table S3. Baseline characteristics of excluded and included participants. Table S4. Subgroup analysis for the association between time-weighted cumulative AIP and risk of myocardial infarction. Table S5. Subgroup analysis for the association between duration of high AIP exposure and risk of myocardial infarction. Table S6. Subgroup analysis for the association between time course of AIP accumulation and risk of myocardial infarction. Table S7. Reclassification and discrimination statistics for risk of MI by time-weighted cumulative AIP. [file 12933_2023_1936_MOESM1_ESM.docx]

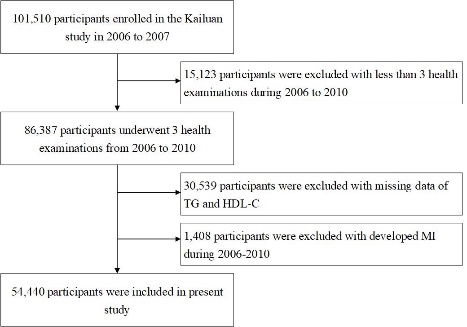


**Figure. S1 The flowchart of the study**

Abbreviation: MI, myocardial infarction; TG, triglyceride; HDL-C, high-density lipoprotein cholesterol; AIP, atherogenic index of plasma.

**Table S1. Baseline characteristics of participants stratified by duration of high AIP exposure**

| Characteristics | 0 year | 2 years | 4 years | 6 years | *P* value |
| --- | --- | --- | --- | --- | --- |
| No. of participants | 30,893 | 11,526 | 6,758 | 5,263 |  |
| Age, year | 49.90 (41.93-57.11) | 49.28 (41.76-56.13) | 49.13 (41.87-55.82) | 48.88 (41.81-55.40) | <0.0001 |
| Male, n (%) | 22,390 (72.48) | 9,230 (80.08) | 5,585 (82.64) | 4,447 (84.50) | <0.0001 |
| High school or above, n (%) | 2,621 (8.48) | 773 (6.71) | 503 (7.44) | 430 (8.17) | <0.0001 |
| Income >800 RMB/month, n (%) | 4,648 (15.05) | 1,678 (14.56) | 1,058 (15.66) | 814 (15.47) | 0.19 |
| Active physical activity, n (%) | 27,348 (88.52) | 9,988 (86.66) | 5,876 (86.95) | 45,78 (86.98) | <0.0001 |
| Current smoking, n (%) | 9,613 (31.12) | 4,054 (35.17) | 2,641 (39.08) | 2,083 (39.58) | <0.0001 |
| Current drinking, n (%) | 11,214 (36.30) | 4,659 (40.42) | 2,915 (43.13) | 2,362 (44.88) | <0.0001 |
| BMI, kg/m^2^ | 24.03 (21.89-26.33) | 25.39 (23.36-27.64) | 26.22 (24.22-28.34) | 26.79 (24.91-28.73) | <0.0001 |
| SBP, mmHg | 121.30 (110.70-140) | 129.30 (119.30-140) | 130 (120-140.70) | 130 (120-142.70) | <0.0001 |
| DBP, mmHg | 80 (72.70-90) | 80 (79.30-90) | 81 (79.30-90) | 82 (80-90) | <0.0001 |
| Hypertension, n (%) | 10,794 (34.94) | 4,999 (43.37) | 3,138 (46.43) | 2,544 (48.34) | <0.0001 |
| Hyperlipidemia, n (%) | 1,906 (6.17) | 1,081 (9.38) | 774 (11.45) | 695 (13.21) | <0.0001 |
| Diabetes Mellitus, n (%) | 5,173 (16.74) | 4,776 (41.44) | 4,116 (60.91) | 4,640 (88.16) | <0.0001 |
| Antihypertensive drugs, n (%) | 2,409 (7.80) | 1,257 (10.91) | 831 (12.30) | 704 (13.38) | <0.0001 |
| Antidiabetic drugs, n (%) | 551 (1.78) | 256 (2.22) | 171 (2.53) | 150 (2.85) | <0.0001 |
| Lipid-lowering drugs, n (%) | 215 (0.70) | 99 (0.86) | 90 (1.33) | 82 (1.56) | <0.0001 |
| FPG, mmol/L | 5.01 (4.59-5.52) | 5.13 (4.67-5.73) | 5.20 (4.73-5.87) | 5.32 (4.81-6.00) | <0.0001 |
| hs-CRP, mg/dL | 0.67 (0.24-1.89) | 0.81 (0.30-2.30) | 1.00 (0.40-2.53) | 1.10 (0.50-2.90) | <0.0001 |
| eGFR, mL/min/1.73m^2^ | 82.58 (69.39-96.95) | 82.5 (68.76-97.6) | 82.77 (69.8-97.28) | 84.01 (70.82-97.63) | <0.0001 |
| TC, mmol/L | 4.83 (4.23-5.46) | 4.91 (4.22-5.58) | 5.03 (4.37-5.77) | 5.11 (4.43-5.82) | <0.0001 |
| LDL-C, mmol/L | 2.27 (1.75-2.75) | 2.37 (1.89-2.83) | 2.33 (1.83-2.82) | 2.18 (1.61-2.74) | <0.0001 |

Abbreviation: AIP, atherogenic index of plasma; BMI, body mass index; SBP, systolic blood pressure; DBP, diastolic blood pressure; FPG, fasting plasma glucose; hs-CRP, high-sensitivity C-reactive protein; eGFR, estimated glomerular filtration rate; TC, total cholesterol; LDL-C, low-density lipoprotein cholesterol.

**Table S2. Baseline characteristics of participants stratified by time course of AIP accumulation**

| Characteristics | Low time-weighted cumulative AIP with positive slope | Low time-weighted cumulative AIP with negative slope | High time-weighted cumulative AIP with positive slope | High time-weighted cumulative AIP with negative slope | *P* value |
| --- | --- | --- | --- | --- | --- |
| No. of participants | 13,192 | 14,028 | 14,183 | 13,037 |  |
| Age, year | 48.89 (40.87-56.18) | 50.68 (42.77-57.91) | 48.3 (40.85-54.83) | 50.45 (42.83-57.3) | <0.0001 |
| Male, n (%) | 9,634 (73.03) | 9,889 (70.49) | 11,447 (80.71) | 10,682 (81.94) | <0.0001 |
| High school or above, n (%) | 1,073 (8.13) | 1,251 (8.92) | 1,050 (7.40) | 953 (7.31) | <0.0001 |
| Income >800 RMB/month, n (%) | 1,970 (14.93) | 2,174 (15.50) | 2,054 (14.48) | 2,000 (15.34) | 0.08 |
| Active physical activity, n (%) | 11,475 (86.98) | 12,547 (89.44) | 12,469 (87.92) | 11,299 (86.67) | <0.0001 |
| Current smoking, n (%) | 4,342 (32.91) | 4,240 (30.23) | 5,084 (35.85) | 4,725 (36.24) | <0.0001 |
| Current drinking, n (%) | 4,993 (37.85) | 5,097 (36.33) | 5,681 (40.05) | 5,379 (41.26) | <0.0001 |
| BMI, kg/m^2^ | 23.67 (21.60-25.95) | 23.94 (21.80-26.21) | 25.77 (23.78-27.91) | 26.08 (24.03-28.23) | <0.0001 |
| SBP, mmHg | 120.70 (110-137.30) | 121.30 (110.70-140) | 129.30 (119.30-140) | 130 (120-142) | <0.0001 |
| DBP, mmHg | 80 (71.70-87.30) | 80 (72.30-89.30) | 80 (79.30-90) | 81.30 (80-90) | <0.0001 |
| Hypertension, n (%) | 4,135 (31.34) | 4,883 (34.81) | 6,117 (43.13) | 6,340 (48.63) | <0.0001 |
| Hyperlipidemia, n (%) | 637 (4.83) | 931 (6.64) | 1,272 (8.97) | 1,616 (12.40) | <0.0001 |
| Diabetes Mellitus, n (%) | 1,798 (13.63) | 2,935 (20.92) | 5,446 (38.40) | 8,526 (65.40) | <0.0001 |
| Antihypertensive drugs, n (%) | 869 (6.59) | 1,113 (7.93) | 1,516 (10.69) | 1,703 (13.06) | <0.0001 |
| Antidiabetic drugs, n (%) | 180 (1.36) | 274 (1.95) | 297 (2.09) | 377 (2.89) | <0.0001 |
| Lipid-lowering drugs, n (%) | 85 (0.64) | 99 (0.71) | 140 (0.99) | 162 (1.24) | <0.0001 |
| FPG, mmol/L | 5.00 (4.56-5.50) | 5.00 (4.58-5.50) | 5.16 (4.72-5.77) | 5.21 (4.71-5.90) | <0.0001 |
| hs-CRP, mg/dL | 0.70 (0.23-2.07) | 0.60 (0.22-1.74) | 0.90 (0.36-2.50) | 0.89 (0.38-2.23) | <0.0001 |
| eGFR, mL/min/1.73m^2^ | 85.9 (72.55-99.52) | 80.91 (68.71-95.15) | 84.38 (70.71-98.29) | 79.13 (66.12-94.9) | <0.0001 |
| TC, mmol/L | 4.82 (4.23-5.44) | 4.82 (4.23-5.45) | 5.02 (4.37-5.71) | 4.93 (4.21-5.65) | <0.0001 |
| LDL-C, mmol/L | 2.22 (1.66-2.74) | 2.23 (1.74-2.74) | 2.32 (1.85-2.8) | 2.36 (1.86-2.83) | <0.0001 |

Continuous variables were reported as median (IQR). Categorical variables were reported as N(%).

Abbreviation: AIP, atherogenic index of plasma; BMI, body mass index; SBP, systolic blood pressure; DBP, diastolic blood pressure; FPG, fasting plasma glucose; hs-CRP, high-sensitivity C-reactive protein; eGFR, estimated glomerular filtration rate; TC, total cholesterol; LDL-C, low-density lipoprotein cholesterol.

**Table S3. Baseline characteristics of excluded and included participants**

| Characteristic | Excluded | Included | *P* value |
| --- | --- | --- | --- |
| No. of participants | 47,070 | 54,440 |  |
| Age, year | 54.70 (48.16-63.1) | 49.58 (41.87-56.57) | <0.0001 |
| Male, n (%) | 39,458 (83.83) | 41,652 (76.51) | 0.003 |
| High school or above, n (%) | 2,443 (5.43) | 4,327 (8.24) | <0.0001 |
| Income >800 RMB/month, n (%) | 5,817 (12.93) | 8,198 (15.62) | <0.0001 |
| Active physical activity, n (%) | 41,032 (87.17) | 47,790 (87.78) | 0.003 |
| Current smoking, n (%) | 15,404 (33.96) | 18,391 (34.74) | 0.01 |
| Current drinking, n (%) | 15,502 (34.16) | 21,150 (39.95) | <0.0001 |
| BMI, kg/m^2^ | 24.81 (22.6-27.18) | 24.91 (22.66-27.27) | 0.003 |
| SBP, mmHg | 130 (120-149.30) | 125.3 (115-140) | <0.0001 |
| DBP, mmHg | 81 (79.30-90) | 80 (76.70-90) | <0.0001 |
| Hypertension, n (%) | 23,178 (49.24) | 21,475 (39.45) | <0.0001 |
| Hyperlipidemia, n (%) | 5,033 (10.69) | 4,456 (8.19) | <0.0001 |
| Diabetes Mellitus, n (%) | 17,688 (37.58) | 18,705 (34.36) | <0.0001 |
| Antihypertensive drugs, n (%) | 6,113 (12.99) | 5,201 (9.55) | <0.0001 |
| Antidiabetic drugs, n (%) | 1,353 (2.87) | 1,128 (2.07) | <0.0001 |
| Lipid-lowering drugs, n (%) | 477 (1.01) | 486 (0.89) | 0.05 |
| FPG, mmol/L | 5.16 (4.70-5.80) | 5.10 (4.64-5.66) | <0.0001 |
| hs-CRP, mg/dL | 0.90 (0.33-2.23) | 0.78 (0.30-2.20) | <0.0001 |
| eGFR, mL/min/1.73m^2^ | 78.3 (65.13-92.69) | 82.72 (69.4-97.21) | <0.0001 |
| TC, mmol/L | 4.97 (4.30-5.62) | 4.90 (4.26-5.56) | <0.0001 |
| LDL-C, mmol/L | 2.40 (1.88-2.90) | 2.30 (1.77-2.79) | <0.0001 |

Continuous variables were reported as median (IQR). Categorical variables were reported as N(%).

Abbreviation: AIP, atherogenic index of plasma; BMI, body mass index; SBP, systolic blood pressure; DBP, diastolic blood pressure; FPG, fasting plasma glucose; hs-CRP, high-sensitivity C-reactive protein; eGFR, estimated glomerular filtration rate; TC, total cholesterol; LDL-C, low-density lipoprotein cholesterol.

**Table S4. Subgroup analysis for the association between time-weighted cumulative AIP and risk of myocardial infarction**

| Subgroup | Group | Cases,  n (%) | Model1 | | Model2 | | Model3 | | Model4 | |
| --- | --- | --- | --- | --- | --- | --- | --- | --- | --- | --- |
|  |  |  | HR (95% CI) | *P* _inter_ | HR (95% CI) | *P* _inter_ | HR (95% CI) | *P* _inter_ | HR (95% CI) | *P* _inter_ |
| Age, years |  |  |  | 0.32 |  | 0.72 |  | 0.64 |  | 0.63 |
| ≤60 | Q1 | 65 (0.59) | Reference |  | Reference |  | Reference |  | Reference |  |
|  | Q2 | 103 (0.92) | 1.57(1.15-2.14) |  | 1.47(1.07-2.00) |  | 1.27(0.93-1.74) |  | 1.28(0.93-1.75) |  |
|  | Q3 | 149 (1.32) | 2.24(1.67-3.00) |  | 1.99(1.48-2.66) |  | 1.53(1.13-2.06) |  | 1.56(1.16-2.11) |  |
|  | Q4 | 201 (1.71) | 2.92(2.21-3.86) |  | 2.49(1.88-3.29) |  | 1.64(1.20-2.23) |  | 1.66(1.21-2.26) |  |
| >60 | Q1 | 43 (1.65) | Reference |  | Reference |  | Reference |  | Reference |  |
|  | Q2 | 65 (2.64) | 1.61(1.10-2.37) |  | 1.64(1.12-2.41) |  | 1.57(1.06-2.31) |  | 1.54(1.04-2.27) |  |
|  | Q3 | 76 (3.26) | 2.01(1.38-2.92) |  | 2.06(1.42-3.00) |  | 1.89(1.28-2.79) |  | 1.90(1.28-2.80) |  |
|  | Q4 | 64 (3.39) | 2.09(1.42-3.07) |  | 2.19(1.48-3.22) |  | 1.86(1.20-2.88) |  | 1.91(1.24-2.95) |  |
| Sex |  |  |  | 0.17 |  | 0.75 |  | 0.74 |  | 0.75 |
| Male | Q1 | 96 (1.02) | Reference |  | Reference |  | Reference |  | Reference |  |
|  | Q2 | 155 (1.53) | 1.50(1.17-1.94) |  | 1.61(1.25-2.08) |  | 1.44(1.12-1.86) |  | 1.44(1.11-1.86) |  |
|  | Q3 | 203 (1.88) | 1.85(1.45-2.36) |  | 2.10(1.65-2.68) |  | 1.71(1.33-2.20) |  | 1.73(1.35-2.23) |  |
|  | Q4 | 241 (2.13) | 2.10(1.66-2.66) |  | 2.59(2.04-3.29) |  | 1.84(1.41-2.40) |  | 1.87(1.43-2.43) |  |
| Female | Q1 | 12 (0.28) | Reference |  | Reference |  | Reference |  | Reference |  |
|  | Q2 | 13 (0.37) | 1.31(0.60-2.88) |  | 1.09(0.50-2.40) |  | 0.93(0.42-2.07) |  | 0.90(0.41-2.01) |  |
|  | Q3 | 22 (0.78) | 2.75(1.36-5.56) |  | 1.98(0.98-4.03) |  | 1.54(0.74-3.22) |  | 1.45(0.69-3.04) |  |
|  | Q4 | 24 (1.05) | 3.73(1.87-7.46) |  | 2.37(1.17-4.77) |  | 1.76(0.81-3.84) |  | 1.71(0.78-3.73) |  |
| BMI, kg/m^2^ |  |  |  | 0.46 |  | 0.51 |  | 0.53 |  | 0.53 |
| ≤24 | Q1 | 76 (0.76) | Reference |  | Reference |  | Reference |  | Reference |  |
|  | Q2 | 73 (0.96) | 1.25(0.91-1.73) |  | 1.28(0.93-1.77) |  | 1.25(0.91-1.73) |  | 1.24(0.90-1.71) |  |
|  | Q3 | 84 (1.38) | 1.81(1.33-2.47) |  | 1.86(1.36-2.54) |  | 1.76(1.28-2.42) |  | 1.77(1.29-2.43) |  |
|  | Q4 | 71 (1.64) | 2.15(1.56-2.97) |  | 2.35(1.69-3.25) |  | 2.17(1.52-3.11) |  | 2.22(1.56-3.17) |  |
| >24 | Q1 | 32 (0.87) | Reference |  | Reference |  | Reference |  | Reference |  |
|  | Q2 | 95 (1.59) | 1.83(1.23-2.73) |  | 1.86(1.24-2.77) |  | 1.86(1.24-2.77) |  | 1.86(1.24-2.77) |  |
|  | Q3 | 141 (1.87) | 2.16(1.47-3.17) |  | 2.25(1.53-3.30) |  | 2.25(1.53-3.30) |  | 2.25(1.53-3.30) |  |
|  | Q4 | 194 (2.09) | 2.42(1.66-3.51) |  | 2.63(1.81-3.83) |  | 2.63(1.81-3.83) |  | 2.63(1.81-3.83) |  |
| Hypertension |  |  |  | 0.74 |  | 1.00 |  | 0.99 |  | 0.98 |
| No | Q1 | 56 (0.58) | Reference |  | Reference |  | Reference |  | Reference |  |
|  | Q2 | 73 (0.86) | 1.50(1.06-2.12) |  | 1.51(1.07-2.14) |  | 1.34(0.94-1.90) |  | 1.30(0.92-1.86) |  |
|  | Q3 | 89 (1.18) | 2.05(1.47-2.87) |  | 2.04(1.46-2.85) |  | 1.68(1.19-2.37) |  | 1.68(1.19-2.38) |  |
|  | Q4 | 100 (1.39) | 2.42(1.74-3.36) |  | 2.47(1.78-3.44) |  | 1.70(1.17-2.48) |  | 1.78(1.23-2.59) |  |
| Yes | Q1 | 52 (1.34) | Reference |  | Reference |  | Reference |  | Reference |  |
|  | Q2 | 95 (1.85) | 1.39(0.99-1.95) |  | 1.48(1.06-2.08) |  | 1.41(1.00-1.98) |  | 1.43(1.01-2.01) |  |
|  | Q3 | 136 (2.24) | 1.68(1.22-2.32) |  | 1.92(1.39-2.64) |  | 1.71(1.23-2.38) |  | 1.74(1.25-2.42) |  |
|  | Q4 | 165 (2.58) | 1.94(1.42-2.65) |  | 2.34(1.71-3.20) |  | 1.92(1.36-2.71) |  | 1.91(1.36-2.70) |  |
| Diabetes | |  |  | 0.32 |  | 0.50 |  | 0.61 |  | 0.14 |
| No | Q1 | 91 (0.71) | Reference |  | Reference |  | Reference |  | Reference |  |
|  | Q2 | 140 (1.12) | 1.58(1.21-2.06) |  | 1.58(1.22-2.06) |  | 1.42(1.09-1.86) |  | 1.35(1.04-1.76) |  |
|  | Q3 | 176 (1.45) | 2.04(1.58-2.62) |  | 2.07(1.61-2.67) |  | 1.71(1.31-2.22) |  | 1.80(1.39-2.32) |  |
|  | Q4 | 211 (1.78) | 2.51(1.96-3.21) |  | 2.67(2.09-3.43) |  | 1.93(1.46-2.54) |  | 1.94(1.48-2.54) |  |
| Yes | Q1 | 17 (2.05) | Reference |  | Reference |  | Reference |  | Reference |  |
|  | Q2 | 28 (2.43) | 1.19(0.65-2.17) |  | 1.25(0.69-2.29) |  | 1.23(0.67-2.25) |  | 1.44(0.75-2.75) |  |
|  | Q3 | 49 (3.36) | 1.66(0.95-2.87) |  | 1.76(1.01-3.05) |  | 1.70(0.96-3.00) |  | 1.25(0.65-2.38) |  |
|  | Q4 | 54 (3.06) | 1.51(0.88-2.61) |  | 1.68(0.97-2.90) |  | 1.60(0.89-2.89) |  | 1.43(0.74-2.74) |  |
| Hyperlipidemia |  |  |  | 0.08 |  | 0.16 |  | 0.16 |  | 0.63 |
| No | Q1 | 94 (0.72) | Reference |  | Reference |  | Reference |  | Reference |  |
|  | Q2 | 140 (1.10) | 1.53(1.18-1.98) |  | 1.53(1.18-1.99) |  | 1.36(1.05-1.77) |  | 1.40(1.06-1.85) |  |
|  | Q3 | 192 (1.56) | 2.15(1.68-2.76) |  | 2.18(1.71-2.80) |  | 1.78(1.38-2.30) |  | 1.75(1.32-2.31) |  |
|  | Q4 | 217 (1.81) | 2.51(1.97-3.20) |  | 2.67(2.09-3.41) |  | 1.91(1.46-2.51) |  | 1.69(1.20-2.38) |  |
| Yes | Q1 | 14 (2.21) | Reference |  | Reference |  | Reference |  | Reference |  |
|  | Q2 | 28 (2.99) | 1.35(0.71-2.57) |  | 1.41(0.74-2.68) |  | 1.44(0.75-2.75) |  | 1.40(0.81-2.39) |  |
|  | Q3 | 33 (2.61) | 1.18(0.63-2.21) |  | 1.26(0.67-2.35) |  | 1.23(0.65-2.35) |  | 1.81(1.10-2.97) |  |
|  | Q4 | 48 (2.96) | 1.34(0.74-2.43) |  | 1.50(0.83-2.74) |  | 1.41(0.74-2.71) |  | 2.05(1.27-3.32) |  |
| Antihypertensive drugs | |  |  | 0.89 |  | 0.92 |  | 0.89 |  | 0.60 |
| No | Q1 | 104 (0.78) | Reference |  | Reference |  | Reference |  | Reference |  |
|  | Q2 | 160 (1.20) | 1.55(1.21-1.98) |  | 1.55(1.21-1.99) |  | 1.38(1.08-1.77) |  | 1.42(1.09-1.86) |  |
|  | Q3 | 216 (1.62) | 2.10(1.66-2.65) |  | 2.12(1.68-2.68) |  | 1.73(1.35-2.20) |  | 1.73(1.33-2.25) |  |
|  | Q4 | 250 (1.89) | 2.45(1.95-3.08) |  | 2.59(2.06-3.27) |  | 1.85(1.43-2.39) |  | 1.97(1.49-2.60) |  |
| Yes | Q1 | 4 (2.00) | Reference |  | Reference |  | Reference |  | Reference |  |
|  | Q2 | 8 (3.15) | 1.59(0.48-5.28) |  | 1.63(0.49-5.41) |  | 1.89(0.55-6.44) |  | 1.20(0.65-2.21) |  |
|  | Q3 | 9 (2.98) | 1.50(0.46-4.87) |  | 1.54(0.47-5.01) |  | 1.57(0.46-5.35) |  | 1.72(0.97-3.03) |  |
|  | Q4 | 15 (4.03) | 2.04(0.68-6.15) |  | 2.15(0.71-6.51) |  | 2.34(0.70-7.83) |  | 1.59(0.88-2.88) |  |
| Antidiabetic drugs |  |  |  | 0.77 |  | 0.73 |  | 0.79 |  | 0.85 |
| No | Q1 | 89 (0.76) | Reference |  | Reference |  | Reference |  | Reference |  |
|  | Q2 | 124 (1.15) | 1.53(1.16-2.00) |  | 1.55(1.18-2.04) |  | 1.42(1.08-1.87) |  | 1.38(1.07-1.77) |  |
|  | Q3 | 132 (1.48) | 1.96(1.50-2.56) |  | 2.03(1.55-2.66) |  | 1.78(1.34-2.34) |  | 1.74(1.37-2.22) |  |
|  | Q4 | 59 (1.37) | 1.81(1.30-2.52) |  | 2.03(1.46-2.83) |  | 1.74(1.23-2.45) |  | 1.87(1.45-2.41) |  |
| Yes | Q1 | 19 (1.02) | Reference |  | Reference |  | Reference |  | Reference |  |
|  | Q2 | 44 (1.54) | 1.51(0.88-2.59) |  | 1.51(0.88-2.58) |  | 1.39(0.81-2.38) |  | 1.99(0.57-6.89) |  |
|  | Q3 | 93 (1.99) | 1.95(1.19-3.19) |  | 1.99(1.22-3.26) |  | 1.71(1.04-2.82) |  | 1.64(0.48-5.64) |  |
|  | Q4 | 206 (2.22) | 2.18(1.36-3.48) |  | 2.37(1.48-3.80) |  | 1.95(1.21-3.14) |  | 2.41(0.71-8.18) |  |
| Lipid-lowering drugs | |  |  | 0.89 |  | 0.93 |  | 0.94 |  | 0.92 |
| No | Q1 | 107 (0.79) | Reference |  | Reference |  | Reference |  | Reference |  |
|  | Q2 | 166 (1.23) | 1.56(1.22-1.99) |  | 1.57(1.23-2.00) |  | 1.40(1.09-1.79) |  | 1.39(1.09-1.78) |  |
|  | Q3 | 223 (1.65) | 2.10(1.67-2.64) |  | 2.12(1.69-2.68) |  | 1.72(1.35-2.18) |  | 1.74(1.37-2.21) |  |
|  | Q4 | 262 (1.95) | 2.48(1.98-3.11) |  | 2.64(2.10-3.30) |  | 1.87(1.45-2.40) |  | 1.90(1.48-2.44) |  |
| Yes | Q1 | 1 (1.47) | Reference |  | Reference |  | Reference |  | Reference |  |
|  | Q2 | 2 (1.72) | 1.17(0.11-12.86) |  | 1.19(0.11-13.22) |  | 1.25(0.10-15.28) |  | 1.25(0.10-15.32) |  |
|  | Q3 | 2 (1.69) | 1.16(0.10-12.76) |  | 1.17(0.11-12.99) |  | 1.29(0.11-15.63) |  | 1.61(0.12-20.75) |  |
|  | Q4 | 3 (1.63) | 1.11(0.12-10.69) |  | 1.24(0.13-12.29) |  | 1.14(0.10-12.71) |  | 1.14(0.10-12.70) |  |
| LDL-C, mmol/L |  |  |  | 0.23 |  | 0.30 |  | 0.42 |  | 0.41 |
| ≤2.6 | Q1 | 67 (0.68) | Reference |  | Reference |  | Reference |  | Reference |  |
|  | Q2 | 95 (1.05) | 1.54(1.13-2.11) |  | 1.55(1.13-2.12) |  | 1.38(1.01-1.90) |  | 1.37(1.00-1.88) |  |
|  | Q3 | 138 (1.58) | 2.32(1.74-3.11) |  | 2.34(1.75-3.14) |  | 1.87(1.38-2.54) |  | 1.88(1.39-2.55) |  |
|  | Q4 | 171 (1.88) | 2.77(2.09-3.67) |  | 2.87(2.16-3.82) |  | 1.92(1.39-2.65) |  | 1.92(1.39-2.64) |  |
| >2.6 | Q1 | 41 (1.08) | Reference |  | Reference |  | Reference |  | Reference |  |
|  | Q2 | 73 (1.59) | 1.48(1.01-2.17) |  | 1.49(1.02-2.18) |  | 1.42(0.96-2.08) |  | 1.43(0.97-2.10) |  |
|  | Q3 | 87 (1.78) | 1.66(1.14-2.40) |  | 1.68(1.16-2.44) |  | 1.52(1.03-2.22) |  | 1.55(1.05-2.27) |  |
|  | Q4 | 94 (2.10) | 1.96(1.36-2.83) |  | 2.16(1.50-3.13) |  | 1.81(1.21-2.70) |  | 1.87(1.25-2.78) |  |

Abbreviation: AIP, atherogenic index of plasma; LDL-C, low-density lipoprotein cholesterol.

Model 1: unadjusted;

Model 2: adjusted for age and sex;

Model 3: Model 2 plus further adjusted for education, income, smoking status, drinking status, body mass index, systolic blood pressure, diastolic blood pressure, fasting plasma glucose, history of hypertension, hyperlipidemia, diabetes at baseline;

Model 4: Model 3 plus further adjusted for high sensitivity C-reactive protein, estimated glomerular filtration rate, total cholesterol, low-density lipoprotein cholesterol level, antihypertensive drugs, antidiabetic drugs, and lipid-lowering drugs at baseline;

*P* _inter_: *P* value for interaction.

**Table S5. Subgroup analysis for the association between duration of high AIP exposure and risk of myocardial infarction**

| Subgroup | Group | Cases,  n (%) | Model1 | | Model2 | | Model3 | | Model4 | |
| --- | --- | --- | --- | --- | --- | --- | --- | --- | --- | --- |
|  |  |  | HR (95% CI) | *P* _inter_ | HR (95% CI) | *P* _inter_ | HR (95% CI) | *P* _inter_ | HR (95% CI) | *P* _inter_ |
| Age, years |  |  |  | 0.07 |  | 0.20 |  | 0.20 |  | 0.23 |
| ≤60 | 0 year | 202 (0.81) | Reference |  | Reference |  | Reference |  | Reference |  |
|  | 2 years | 130 (1.33) | 1.65(1.33-2.06) |  | 1.52(1.22-1.90) |  | 1.28(1.02-1.61) |  | 1.33(1.06-1.67) |  |
|  | 4 years | 100 (1.74) | 2.17(1.71-2.76) |  | 1.93(1.52-2.46) |  | 1.46(1.12-1.90) |  | 1.51(1.16-1.96) |  |
|  | 6 years | 86 (1.88) | 2.35(1.82-3.02) |  | 2.06(1.60-2.66) |  | 1.41(1.05-1.89) |  | 1.41(1.05-1.89) |  |
| >60 | 0 year | 126 (2.17) | Reference |  | Reference |  | Reference |  | Reference |  |
|  | 2 years | 69 (3.87) | 1.80(1.34-2.42) |  | 1.82(1.36-2.45) |  | 1.63(1.20-2.21) |  | 1.68(1.24-2.29) |  |
|  | 4 years | 33 (3.24) | 1.51(1.03-2.21) |  | 1.56(1.06-2.29) |  | 1.34(0.89-2.03) |  | 1.42(0.94-2.15) |  |
|  | 6 years | 20 (2.94) | 1.36(0.85-2.18) |  | 1.41(0.88-2.26) |  | 1.17(0.70-1.95) |  | 1.26(0.75-2.12) |  |
| Sex |  |  |  | 0.20 |  | 0.74 |  | 0.71 |  | 0.69 |
| Male | 0 year | 294 (1.31) | Reference |  | Reference |  | Reference |  | Reference |  |
|  | 2 years | 185 (2.00) | 1.53(1.27-1.84) |  | 1.72(1.43-2.07) |  | 1.48(1.22-1.79) |  | 1.52(1.26-1.84) |  |
|  | 4 years | 120 (2.15) | 1.64(1.33-2.03) |  | 1.92(1.55-2.38) |  | 1.49(1.18-1.88) |  | 1.54(1.22-1.94) |  |
|  | 6 years | 96 (2.16) | 1.66(1.31-2.08) |  | 1.99(1.58-2.52) |  | 1.44(1.10-1.88) |  | 1.46(1.12-1.91) |  |
| Female | 0 year | 34 (0.40) | Reference |  | Reference |  | Reference |  | Reference |  |
|  | 2 years | 14 (0.61) | 1.52(0.82-2.83) |  | 1.26(0.68-2.35) |  | 1.08(0.57-2.05) |  | 1.07(0.56-2.04) |  |
|  | 4 years | 13 (1.11) | 2.79(1.47-5.29) |  | 1.94(1.02-3.70) |  | 1.74(0.87-3.48) |  | 1.78(0.89-3.58) |  |
|  | 6 years | 10 (1.23) | 3.08(1.52-6.24) |  | 2.12(1.04-4.30) |  | 1.67(0.75-3.74) |  | 1.73(0.77-3.90) |  |
| BMI, kg/m^2^ |  |  |  | 0.67 |  | 0.73 |  | 0.80 |  | 0.83 |
| ≤24 | 0 year | 172 (0.90) | Reference |  | Reference |  | Reference |  | Reference |  |
|  | 2 years | 71 (1.37) | 1.52(1.15-2.00) |  | 1.58(1.20-2.08) |  | 1.49(1.12-1.99) |  | 1.56(1.17-2.08) |  |
|  | 4 years | 35 (1.50) | 1.67(1.16-2.40) |  | 1.78(1.23-2.56) |  | 1.71(1.16-2.51) |  | 1.77(1.20-2.59) |  |
|  | 6 years | 26 (1.88) | 2.10(1.39-3.17) |  | 2.27(1.50-3.44) |  | 2.09(1.32-3.30) |  | 2.17(1.37-3.43) |  |
| >24 | 0 year | 156 (1.32) | Reference |  | Reference |  | Reference |  | Reference |  |
|  | 2 years | 128 (2.02) | 1.53(1.21-1.93) |  | 1.62(1.29-2.05) |  | 1.62(1.29-2.05) |  | 1.62(1.29-2.05) |  |
|  | 4 years | 98 (2.21) | 1.68(1.30-2.16) |  | 1.80(1.40-2.32) |  | 1.80(1.40-2.32) |  | 1.80(1.40-2.32) |  |
|  | 6 years | 80 (2.06) | 1.57(1.20-2.05) |  | 1.71(1.30-2.25) |  | 1.71(1.30-2.25) |  | 1.71(1.30-2.25) |  |
| Hypertension |  |  |  | 0.13 |  | 0.42 |  | 0.50 |  | 0.43 |
| No | 0 year | 143 (0.71) | Reference |  | Reference |  | Reference |  | Reference |  |
|  | 2 years | 77 (1.18) | 1.65(1.25-2.18) |  | 1.70(1.28-2.24) |  | 1.49(1.12-1.99) |  | 1.55(1.16-2.06) |  |
|  | 4 years | 55 (1.52) | 2.14(1.57-2.92) |  | 2.20(1.61-3.01) |  | 1.72(1.22-2.42) |  | 1.79(1.27-2.51) |  |
|  | 6 years | 43 (1.58) | 2.23(1.59-3.14) |  | 2.29(1.63-3.23) |  | 1.67(1.12-2.49) |  | 1.85(1.24-2.77) |  |
| Yes | 0 year | 185 (1.71) | Reference |  | Reference |  | Reference |  | Reference |  |
|  | 2 years | 122 (2.44) | 1.43(1.13-1.79) |  | 1.56(1.24-1.96) |  | 1.41(1.11-1.78) |  | 1.43(1.13-1.82) |  |
|  | 4 years | 78 (2.49) | 1.46(1.12-1.90) |  | 1.63(1.25-2.12) |  | 1.38(1.04-1.84) |  | 1.39(1.05-1.86) |  |
|  | 6 years | 63 (2.48) | 1.45(1.09-1.93) |  | 1.67(1.26-2.23) |  | 1.35(0.97-1.86) |  | 1.32(0.95-1.83) |  |
| Diabetes | |  |  | 0.16 |  | 0.30 |  | 0.44 |  | 0.59 |
| No | 0 year | 269 (0.94) | Reference |  | Reference |  | Reference |  | Reference |  |
|  | 2 years | 156 (1.52) | 1.61(1.32-1.96) |  | 1.68(1.38-2.05) |  | 1.46(1.19-1.80) |  | 1.51(1.23-1.84) |  |
|  | 4 years | 106 (1.79) | 1.90(1.51-2.37) |  | 2.00(1.60-2.51) |  | 1.58(1.24-2.03) |  | 1.61(1.27-2.05) |  |
|  | 6 years | 87 (1.91) | 2.03(1.59-2.58) |  | 2.16(1.69-2.76) |  | 1.58(1.19-2.10) |  | 1.54(1.17-2.04) |  |
| Yes | 0 year | 59 (2.45) | Reference |  | Reference |  | Reference |  | Reference |  |
|  | 2 years | 43 (3.42) | 1.40(0.95-2.08) |  | 1.46(0.98-2.16) |  | 1.41(0.94-2.12) |  | 1.33(0.84-2.11) |  |
|  | 4 years | 27 (3.25) | 1.33(0.85-2.11) |  | 1.41(0.89-2.23) |  | 1.33(0.82-2.17) |  | 1.31(0.76-2.26) |  |
|  | 6 years | 19 (2.70) | 1.11(0.66-1.86) |  | 1.22(0.73-2.06) |  | 1.16(0.66-2.06) |  | 1.27(0.70-2.30) |  |
| Hyperlipidemia |  |  |  | 0.27 |  | 0.47 |  | 0.57 |  | 0.77 |
| No | 0 year | 282 (0.97) | Reference |  | Reference |  | Reference |  | Reference |  |
|  | 2 years | 165 (1.58) | 1.62(1.34-1.97) |  | 1.69(1.40-2.05) |  | 1.47(1.20-1.79) |  | 1.54(1.22-1.94) |  |
|  | 4 years | 110 (1.84) | 1.90(1.52-2.36) |  | 2.00(1.60-2.49) |  | 1.57(1.23-1.99) |  | 1.40(0.98-1.98) |  |
|  | 6 years | 86 (1.88) | 1.95(1.53-2.48) |  | 2.07(1.62-2.64) |  | 1.51(1.14-1.99) |  | 1.48(0.78-2.81) |  |
| Yes | 0 year | 46 (2.41) | Reference |  | Reference |  | Reference |  | Reference |  |
|  | 2 years | 34 (3.15) | 1.31(0.84-2.03) |  | 1.36(0.88-2.13) |  | 1.29(0.82-2.05) |  | 1.44(1.06-1.97) |  |
|  | 4 years | 23 (2.97) | 1.23(0.75-2.03) |  | 1.33(0.80-2.19) |  | 1.26(0.73-2.18) |  | 1.64(1.20-2.24) |  |
|  | 6 years | 20 (2.88) | 1.19(0.71-2.02) |  | 1.35(0.80-2.30) |  | 1.25(0.69-2.25) |  | 1.49(1.08-2.05) |  |
| Antihypertensive drugs | |  |  | 0.93 |  | 0.90 |  | 0.87 |  | 0.45 |
| No | 0 year | 314 (1.03) | Reference |  | Reference |  | Reference |  | Reference |  |
|  | 2 years | 190 (1.69) | 1.63(1.36-1.95) |  | 1.70(1.41-2.03) |  | 1.46(1.21-1.76) |  | 1.51(1.23-1.86) |  |
|  | 4 years | 127 (1.93) | 1.87(1.52-2.30) |  | 1.96(1.59-2.41) |  | 1.53(1.22-1.91) |  | 1.63(1.27-2.08) |  |
|  | 6 years | 99 (1.94) | 1.88(1.50-2.36) |  | 2.00(1.59-2.51) |  | 1.44(1.11-1.87) |  | 1.62(1.22-2.15) |  |
| Yes | 0 year | 14 (2.54) | Reference |  | Reference |  | Reference |  | Reference |  |
|  | 2 years | 9 (3.52) | 1.39(0.60-3.22) |  | 1.40(0.60-3.22) |  | 1.27(0.53-3.02) |  | 1.48(0.98-2.22) |  |
|  | 4 years | 6 (3.51) | 1.38(0.53-3.60) |  | 1.43(0.55-3.73) |  | 1.58(0.55-4.50) |  | 1.41(0.87-2.30) |  |
|  | 6 years | 7 (4.67) | 1.85(0.75-4.59) |  | 2.01(0.80-5.02) |  | 2.05(0.76-5.52) |  | 1.16(0.66-2.06) |  |
| Antidiabetic drugs |  |  |  | 0.71 |  | 0.71 |  | 0.78 |  | 0.90 |
| No | 0 year | 253 (0.98) | Reference |  | Reference |  | Reference |  | Reference |  |
|  | 2 years | 104 (1.54) | 1.57(1.25-1.97) |  | 1.69(1.34-2.13) |  | 1.56(1.23-1.96) |  | 1.50(1.24-1.80) |  |
|  | 4 years | 37 (1.40) | 1.43(1.01-2.02) |  | 1.57(1.11-2.22) |  | 1.42(1.00-2.02) |  | 1.56(1.25-1.96) |  |
|  | 6 years | 10 (1.61) | 1.65(0.88-3.10) |  | 1.74(0.92-3.28) |  | 1.53(0.81-2.89) |  | 1.48(1.14-1.92) |  |
| Yes | 0 year | 75 (1.45) | Reference |  | Reference |  | Reference |  | Reference |  |
|  | 2 years | 95 (1.99) | 1.35(1.00-1.83) |  | 1.43(1.06-1.94) |  | 1.33(0.98-1.80) |  | 1.38(0.58-3.31) |  |
|  | 4 years | 96 (2.33) | 1.60(1.18-2.16) |  | 1.73(1.28-2.35) |  | 1.53(1.12-2.08) |  | 1.80(0.62-5.20) |  |
|  | 6 years | 96 (2.07) | 1.42(1.05-1.92) |  | 1.61(1.18-2.18) |  | 1.41(1.03-1.93) |  | 1.88(0.67-5.27) |  |
| Lipid-lowering drugs | |  |  | 0.77 |  | 0.76 |  | 0.75 |  | 0.74 |
| No | 0 year | 324 (1.06) | Reference |  | Reference |  | Reference |  | Reference |  |
|  | 2 years | 198 (1.73) | 1.64(1.37-1.96) |  | 1.71(1.43-2.04) |  | 1.46(1.21-1.75) |  | 1.50(1.25-1.81) |  |
|  | 4 years | 133 (1.99) | 1.89(1.55-2.32) |  | 1.99(1.62-2.43) |  | 1.55(1.24-1.93) |  | 1.60(1.28-1.99) |  |
|  | 6 years | 103 (1.99) | 1.89(1.52-2.36) |  | 2.02(1.62-2.53) |  | 1.46(1.13-1.88) |  | 1.50(1.16-1.94) |  |
| Yes | 0 year | 4 (1.86) | Reference |  | Reference |  | Reference |  | Reference |  |
|  | 2 years | 1 (1.01) | 0.54(0.06-4.87) |  | 0.55(0.06-4.97) |  | 0.48(0.05-4.38) |  | 0.38(0.03-4.71) |  |
|  | 4 years | 0 (0.00) | 0.00(0.00-.) |  | 0.00(0.00-.) |  | 0.00(0.00-.) |  | 0.00(0.00-.) |  |
|  | 6 years | 3 (3.66) | 1.98(0.44-8.84) |  | 2.24(0.48-10.48) |  | 2.00(0.39-10.14) |  | 1.89(0.35-10.08) |  |
| LDL-C, mmol/L |  |  |  | 0.09 |  | 0.13 |  | 0.20 |  | 0.23 |
| ≤2.6 | 0 year | 191 (0.90) | Reference |  | Reference |  | Reference |  | Reference |  |
|  | 2 years | 129 (1.73) | 1.92(1.54-2.40) |  | 1.98(1.58-2.47) |  | 1.64(1.29-2.07) |  | 1.66(1.32-2.10) |  |
|  | 4 years | 80 (1.82) | 2.02(1.55-2.62) |  | 2.08(1.60-2.70) |  | 1.53(1.14-2.04) |  | 1.55(1.16-2.06) |  |
|  | 6 years | 71 (1.94) | 2.16(1.64-2.84) |  | 2.22(1.69-2.93) |  | 1.46(1.06-2.03) |  | 1.47(1.07-2.04) |  |
| >2.6 | 0 year | 137 (1.41) | Reference |  | Reference |  | Reference |  | Reference |  |
|  | 2 years | 70 (1.72) | 1.22(0.91-1.62) |  | 1.29(0.97-1.72) |  | 1.20(0.89-1.61) |  | 1.25(0.93-1.68) |  |
|  | 4 years | 53 (2.26) | 1.61(1.17-2.21) |  | 1.75(1.28-2.41) |  | 1.55(1.10-2.18) |  | 1.61(1.14-2.26) |  |
|  | 6 years | 35 (2.23) | 1.60(1.10-2.32) |  | 1.85(1.27-2.68) |  | 1.58(1.05-2.39) |  | 1.62(1.08-2.45) |  |

Abbreviation: AIP, atherogenic index of plasma; LDL-C, low-density lipoprotein cholesterol.

Model 1: unadjusted;

Model 2: adjusted for age and sex;

Model 3: Model 2 plus further adjusted for education, income, smoking status, drinking status, body mass index, systolic blood pressure, diastolic blood pressure, fasting plasma glucose, history of hypertension, hyperlipidemia, diabetes at baseline;

Model 4: Model 3 plus further adjusted for high sensitivity C-reactive protein, estimated glomerular filtration rate, total cholesterol, low-density lipoprotein cholesterol level, antihypertensive drugs, antidiabetic drugs, and lipid-lowering drugs at baseline;

*P* _inter_: *P* value for interaction.

**Table S6. Subgroup analysis for the association between time course of AIP accumulation and risk of myocardial infarction**

| Subgroup | Group | Cases,  n (%) | Model1 | | Model2 | | Model3 | | Model4 | |
| --- | --- | --- | --- | --- | --- | --- | --- | --- | --- | --- |
|  |  |  | HR (95% CI) | *P* _inter_ | HR (95% CI) | *P* _inter_ | HR (95% CI) | *P* _inter_ | HR (95% CI) | *P* _inter_ |
| Age, years |  |  |  | 0.07 |  | 0.13 |  | 0.14 |  | 0.11 |
| ≤60 | Group1 | 78 (0.71) | Reference |  | Reference |  | Reference |  | Reference |  |
|  | Group2 | 90 (0.81) | 1.14(0.84-1.54) |  | 1.18(0.87-1.60) |  | 1.11(0.82-1.50) |  | 1.12(0.83-1.52) |  |
|  | Group3 | 179 (1.45) | 2.05(1.57-2.68) |  | 1.88(1.44-2.45) |  | 1.47(1.12-1.94) |  | 1.46(1.11-1.93) |  |
|  | Group4 | 171 (1.60) | 2.27(1.74-2.96) |  | 2.07(1.58-2.70) |  | 1.40(1.04-1.87) |  | 1.47(1.10-1.98) |  |
| >60 | Group1 | 57 (2.56) | Reference |  | Reference |  | Reference |  | Reference |  |
|  | Group2 | 51 (1.79) | 0.69(0.48-1.01) |  | 0.69(0.47-1.01) |  | 0.69(0.47-1.01) |  | 0.68(0.47-1.00) |  |
|  | Group3 | 54 (2.93) | 1.15(0.79-1.67) |  | 1.19(0.82-1.73) |  | 1.06(0.72-1.57) |  | 1.03(0.70-1.52) |  |
|  | Group4 | 86 (3.63) | 1.42(1.02-1.99) |  | 1.45(1.04-2.03) |  | 1.30(0.90-1.87) |  | 1.38(0.96-1.99) |  |
| Sex |  |  |  | 0.03 |  | 0.15 |  | 0.18 |  | 0.23 |
| Male | Group1 | 125 (1.30) | Reference |  | Reference |  | Reference |  | Reference |  |
|  | Group2 | 126 (1.27) | 0.99(0.77-1.26) |  | 0.88(0.69-1.13) |  | 0.85(0.67-1.10) |  | 0.87(0.68-1.11) |  |
|  | Group3 | 216 (1.89) | 1.46(1.17-1.82) |  | 1.66(1.33-2.08) |  | 1.35(1.07-1.70) |  | 1.33(1.06-1.68) |  |
|  | Group4 | 228 (2.13) | 1.66(1.33-2.06) |  | 1.68(1.35-2.09) |  | 1.25(0.98-1.58) |  | 1.33(1.05-1.69) |  |
| Female | Group1 | 10 (0.28) | Reference |  | Reference |  | Reference |  | Reference |  |
|  | Group2 | 15 (0.36) | 1.30(0.58-2.89) |  | 1.27(0.57-2.83) |  | 1.16(0.52-2.58) |  | 1.11(0.50-2.49) |  |
|  | Group3 | 17 (0.62) | 2.23(1.02-4.86) |  | 1.70(0.78-3.73) |  | 1.42(0.63-3.17) |  | 1.37(0.61-3.08) |  |
|  | Group4 | 29 (1.23) | 4.42(2.15-9.07) |  | 3.11(1.51-6.40) |  | 2.40(1.10-5.23) |  | 2.22(1.01-4.86) |  |
| BMI, kg/m^2^ |  |  |  | 0.78 |  | 0.88 |  | 0.87 |  | 0.85 |
| ≤24 | Group1 | 76 (0.88) | Reference |  | Reference |  | Reference |  | Reference |  |
|  | Group2 | 73 (0.82) | 0.94(0.68-1.30) |  | 0.85(0.61-1.17) |  | 0.83(0.60-1.14) |  | 0.84(0.61-1.16) |  |
|  | Group3 | 79 (1.39) | 1.59(1.16-2.18) |  | 1.68(1.22-2.30) |  | 1.57(1.14-2.16) |  | 1.53(1.11-2.11) |  |
|  | Group4 | 76 (1.61) | 1.85(1.34-2.54) |  | 1.69(1.23-2.32) |  | 1.52(1.08-2.14) |  | 1.66(1.18-2.33) |  |
| >24 | Group1 | 59 (1.31) | Reference |  | Reference |  | Reference |  | Reference |  |
|  | Group2 | 68 (1.32) | 1.01(0.72-1.44) |  | 0.95(0.67-1.35) |  | 0.95(0.67-1.35) |  | 0.95(0.67-1.35) |  |
|  | Group3 | 154 (1.81) | 1.39(1.03-1.88) |  | 1.50(1.11-2.02) |  | 1.50(1.11-2.02) |  | 1.50(1.11-2.02) |  |
|  | Group4 | 181 (2.18) | 1.68(1.25-2.25) |  | 1.62(1.21-2.18) |  | 1.62(1.21-2.18) |  | 1.62(1.21-2.18) |  |
| Hypertension |  |  |  | 0.14 |  | 0.21 |  | 0.21 |  | 0.19 |
| No | Group1 | 64 (0.71) | Reference |  | Reference |  | Reference |  | Reference |  |
|  | Group2 | 65 (0.71) | 1.02(0.72-1.43) |  | 0.92(0.65-1.31) |  | 0.90(0.64-1.28) |  | 0.92(0.65-1.30) |  |
|  | Group3 | 106 (1.31) | 1.87(1.37-2.55) |  | 1.91(1.40-2.60) |  | 1.54(1.11-2.12) |  | 1.54(1.12-2.13) |  |
|  | Group4 | 83 (1.24) | 1.77(1.28-2.45) |  | 1.57(1.13-2.17) |  | 1.15(0.80-1.64) |  | 1.25(0.88-1.79) |  |
| Yes | Group1 | 71 (1.72) | Reference |  | Reference |  | Reference |  | Reference |  |
|  | Group2 | 76 (1.56) | 0.91(0.66-1.25) |  | 0.87(0.63-1.21) |  | 0.86(0.62-1.19) |  | 0.88(0.64-1.22) |  |
|  | Group3 | 127 (2.08) | 1.21(0.91-1.62) |  | 1.38(1.03-1.85) |  | 1.23(0.91-1.66) |  | 1.21(0.90-1.64) |  |
|  | Group4 | 174 (2.74) | 1.61(1.22-2.12) |  | 1.72(1.30-2.26) |  | 1.45(1.08-1.95) |  | 1.52(1.13-2.05) |  |
| Diabetes | |  |  | 0.09 |  | 0.05 |  | 0.04 |  | 0.07 |
| No | Group1 | 118 (0.96) | Reference |  | Reference |  | Reference |  | Reference |  |
|  | Group2 | 113 (0.87) | 0.92(0.71-1.19) |  | 0.84(0.65-1.09) |  | 0.81(0.63-1.05) |  | 0.91(0.71-1.18) |  |
|  | Group3 | 198 (1.56) | 1.64(1.30-2.06) |  | 1.73(1.37-2.17) |  | 1.40(1.10-1.77) |  | 1.42(1.12-1.80) |  |
|  | Group4 | 189 (1.67) | 1.75(1.39-2.20) |  | 1.63(1.29-2.05) |  | 1.19(0.93-1.53) |  | 1.55(1.21-1.98) |  |
| Yes | Group1 | 17 (1.96) | Reference |  | Reference |  | Reference |  | Reference |  |
|  | Group2 | 28 (2.52) | 1.28(0.70-2.35) |  | 1.27(0.70-2.33) |  | 1.30(0.71-2.38) |  | 0.72(0.39-1.33) |  |
|  | Group3 | 35 (2.31) | 1.19(0.67-2.13) |  | 1.27(0.71-2.26) |  | 1.27(0.70-2.30) |  | 0.90(0.51-1.59) |  |
|  | Group4 | 68 (3.99) | 2.06(1.21-3.50) |  | 2.12(1.24-3.60) |  | 2.16(1.24-3.78) |  | 0.85(0.48-1.49) |  |
| Hyperlipidemia |  |  |  | 0.02 |  | 0.07 |  | 0.08 |  | 0.65 |
| No | Group1 | 115 (0.92) | Reference |  | Reference |  | Reference |  | Reference |  |
|  | Group2 | 119 (0.91) | 1.00(0.77-1.29) |  | 0.92(0.71-1.19) |  | 0.90(0.69-1.16) |  | 0.94(0.72-1.24) |  |
|  | Group3 | 197 (1.53) | 1.67(1.33-2.11) |  | 1.76(1.40-2.22) |  | 1.43(1.13-1.82) |  | 1.34(1.02-1.75) |  |
|  | Group4 | 212 (1.86) | 2.04(1.63-2.56) |  | 1.90(1.51-2.38) |  | 1.45(1.13-1.86) |  | 1.45(1.07-1.97) |  |
| Yes | Group1 | 20 (3.14) | Reference |  | Reference |  | Reference |  | Reference |  |
|  | Group2 | 22 (2.36) | 0.75(0.41-1.37) |  | 0.71(0.39-1.30) |  | 0.72(0.39-1.33) |  | 0.72(0.44-1.18) |  |
|  | Group3 | 36 (2.83) | 0.90(0.52-1.56) |  | 0.95(0.55-1.65) |  | 0.91(0.52-1.59) |  | 1.29(0.86-1.95) |  |
|  | Group4 | 45 (2.78) | 0.89(0.52-1.50) |  | 0.91(0.53-1.53) |  | 0.83(0.47-1.46) |  | 1.29(0.87-1.92) |  |
| Antihypertensive drugs | |  |  | 0.79 |  | 0.81 |  | 0.76 |  | 0.05 |
| No | Group1 | 130 (1.00) | Reference |  | Reference |  | Reference |  | Reference |  |
|  | Group2 | 134 (0.97) | 0.98(0.77-1.25) |  | 0.90(0.71-1.15) |  | 0.87(0.69-1.11) |  | 0.82(0.64-1.07) |  |
|  | Group3 | 225 (1.62) | 1.63(1.31-2.02) |  | 1.71(1.37-2.12) |  | 1.39(1.11-1.74) |  | 1.39(1.10-1.77) |  |
|  | Group4 | 241 (1.90) | 1.92(1.55-2.38) |  | 1.78(1.44-2.20) |  | 1.33(1.05-1.68) |  | 1.27(0.99-1.64) |  |
| Yes | Group1 | 5 (2.78) | Reference |  | Reference |  | Reference |  | Reference |  |
|  | Group2 | 7 (2.55) | 0.92(0.29-2.89) |  | 0.91(0.29-2.86) |  | 0.92(0.29-2.91) |  | 1.27(0.69-2.33) |  |
|  | Group3 | 8 (2.69) | 0.97(0.32-2.98) |  | 1.00(0.33-3.07) |  | 0.95(0.30-3.01) |  | 1.25(0.69-2.26) |  |
|  | Group4 | 16 (4.24) | 1.53(0.56-4.18) |  | 1.55(0.57-4.22) |  | 1.45(0.50-4.17) |  | 2.20(1.26-3.86) |  |
| Antidiabetic drugs |  |  |  | 0.19 |  | 0.50 |  | 0.55 |  | 0.81 |
| No | Group1 | 105 (0.92) | Reference |  | Reference |  | Reference |  | Reference |  |
|  | Group2 | 108 (0.97) | 1.06(0.81-1.39) |  | 0.95(0.73-1.24) |  | 0.94(0.72-1.23) |  | 0.89(0.70-1.13) |  |
|  | Group3 | 115 (1.32) | 1.43(1.10-1.87) |  | 1.53(1.17-1.99) |  | 1.36(1.04-1.79) |  | 1.37(1.10-1.72) |  |
|  | Group4 | 76 (1.68) | 1.85(1.37-2.48) |  | 1.63(1.21-2.19) |  | 1.46(1.08-1.97) |  | 1.41(1.12-1.78) |  |
| Yes | Group1 | 30 (1.67) | Reference |  | Reference |  | Reference |  | Reference |  |
|  | Group2 | 33 (1.12) | 0.67(0.41-1.10) |  | 0.68(0.42-1.12) |  | 0.69(0.42-1.14) |  | 0.84(0.26-2.69) |  |
|  | Group3 | 118 (2.17) | 1.29(0.87-1.93) |  | 1.44(0.96-2.16) |  | 1.28(0.85-1.91) |  | 0.89(0.28-2.88) |  |
|  | Group4 | 181 (2.12) | 1.26(0.86-1.86) |  | 1.33(0.90-1.96) |  | 1.17(0.79-1.74) |  | 1.40(0.48-4.08) |  |
| Lipid-lowering drugs | |  |  | 0.74 |  | 0.87 |  | 0.89 |  | 0.83 |
| No | Group1 | 133 (1.01) | Reference |  | Reference |  | Reference |  | Reference |  |
|  | Group2 | 140 (1.01) | 1.00(0.78-1.26) |  | 0.91(0.72-1.15) |  | 0.88(0.70-1.12) |  | 0.90(0.71-1.14) |  |
|  | Group3 | 231 (1.64) | 1.63(1.32-2.02) |  | 1.70(1.37-2.11) |  | 1.38(1.10-1.72) |  | 1.37(1.09-1.70) |  |
|  | Group4 | 254 (1.97) | 1.96(1.59-2.42) |  | 1.81(1.47-2.24) |  | 1.34(1.07-1.69) |  | 1.44(1.14-1.81) |  |
| Yes | Group1 | 2 (2.35) | Reference |  | Reference |  | Reference |  | Reference |  |
|  | Group2 | 1 (1.01) | 0.43(0.04-4.73) |  | 0.47(0.04-5.32) |  | 0.42(0.03-5.11) |  | 0.38(0.03-4.79) |  |
|  | Group3 | 2 (1.43) | 0.62(0.09-4.38) |  | 0.71(0.10-5.16) |  | 0.58(0.07-4.79) |  | 0.74(0.08-6.52) |  |
|  | Group4 | 3 (1.85) | 0.79(0.13-4.70) |  | 0.85(0.14-5.16) |  | 0.79(0.12-5.20) |  | 0.71(0.10-5.04) |  |
| LDL-C, mmol/L |  |  |  | 0.09 |  | 0.10 |  | 0.19 |  | 0.16 |
| ≤2.6 | Group1 | 75 (0.82) | Reference |  | Reference |  | Reference |  | Reference |  |
|  | Group2 | 87 (0.90) | 1.10(0.81-1.50) |  | 1.03(0.75-1.40) |  | 1.02(0.74-1.39) |  | 1.04(0.76-1.42) |  |
|  | Group3 | 150 (1.59) | 1.95(1.48-2.57) |  | 2.02(1.53-2.67) |  | 1.60(1.19-2.13) |  | 1.58(1.18-2.11) |  |
|  | Group4 | 159 (1.90) | 2.34(1.77-3.07) |  | 2.16(1.64-2.84) |  | 1.56(1.15-2.12) |  | 1.65(1.22-2.24) |  |
| >2.6 | Group1 | 60 (1.48) | Reference |  | Reference |  | Reference |  | Reference |  |
|  | Group2 | 54 (1.25) | 0.84(0.58-1.21) |  | 0.74(0.51-1.07) |  | 0.72(0.50-1.05) |  | 0.72(0.50-1.04) |  |
|  | Group3 | 83 (1.75) | 1.18(0.85-1.65) |  | 1.25(0.90-1.75) |  | 1.11(0.79-1.56) |  | 1.10(0.78-1.55) |  |
|  | Group4 | 98 (2.12) | 1.44(1.04-1.98) |  | 1.32(0.96-1.82) |  | 1.11(0.79-1.57) |  | 1.16(0.82-1.64) |  |

Abbreviation: AIP, atherogenic index of plasma; LDL-C, low-density lipoprotein cholesterol.

Groups: Group1, low time-weighted cumulative AIP with positive slope; Group2, low time-weighted cumulative AIP with negative slope; Group3, high time-weighted cumulative AIP with positive slope; Group4, high time-weighted cumulative AIP with negative slope.

Model 1: unadjusted;

Model 2: adjusted for age and sex;

Model 3: Model 2 plus further adjusted for education, income, smoking status, drinking status, body mass index, systolic blood pressure, diastolic blood pressure, fasting plasma glucose, history of hypertension, hyperlipidemia, diabetes at baseline;

Model 4: Model 3 plus further adjusted for high sensitivity C-reactive protein, estimated glomerular filtration rate, total cholesterol, low-density lipoprotein cholesterol level, antihypertensive drugs, antidiabetic drugs, and lipid-lowering drugs at baseline;

*P* _inter_: *P* value for interaction.

Table S7. Reclassification and discrimination statistics for risk of MI by time-weighted cumulative AIP

|  | C statistics | | IDI | | Category-free NRI | |
| --- | --- | --- | --- | --- | --- | --- |
|  | Estimate (95% CI) | *P* value | Estimate (95% CI), % | *P* value | Estimate (95% CI), % | *P* value |
| Conventional model^*^ | 0.73(0.71-0.74) |  | Reference |  | Reference |  |
| Conventional model +cumAIP | 0.74(0.72-0.75) | 0.002 | 0.05(0.001-0.08) | 0.009 | 17.21(10.09-24.33) | <0.0001 |
| Conventional model +LDL-C | 0.73(0.71-0.75) | 0.697 | 0.002(-0.01-0.01) | 0.700 | 2.37(-4.76-9.50) | 0.514 |
| Conventional model + non-HDL | 0.73(0.71-0.75) | 0.279 | 0.01(0.00-0.02) | 0.241 | 4.74(-2.31-11.78) | <0.0001 |
| Conventional model + HDL-C | 0.73(0.71-0.75) | 0.280 | 0.006(0.00-0.02) | 0.257 | 5.12(-1.92-12.17) | <0.0001 |
| Conventional model + TG | 0.73(0.72-0.75) | 0.017 | 0.004(-0.01-0.02) | 0.584 | 16.29(9.17-23.41) | <0.0001 |

Abbreviations: MI, myocardial infarction; cumAIP, time-weighted cumulative atherogenic index of plasma; LDL-C, low-density lipoprotein cholesterol; HDL-C, high-density lipoprotein cholesterol; TG, triglyceride; IDI, integrated discrimination improvement; NRI, net reclassification index.

^*^Conventional model was adjusted for age, sex, education, income, smoking status, drinking status, body mass index, systolic blood pressure, diastolic blood pressure, fasting plasma glucose, high sensitivity C-reactive protein, estimated glomerular filtration rate, total cholesterol, history of hypertension, hyperlipidemia, diabetes, antihypertensive drugs, antidiabetic drugs, and lipid-lowering drugs at baseline.
